# Human rhinovirus-induced inflammatory responses are inhibited by phosphatidylserine containing liposomes

**DOI:** 10.1038/mi.2015.137

**Published:** 2016-02-24

**Authors:** C A Stokes, R Kaur, M R Edwards, M Mondhe, D Robinson, E C Prestwich, R D Hume, C A Marshall, Y Perrie, V B O'Donnell, J L Harwood, I Sabroe, L C Parker

**Affiliations:** 1 Department of Infection, Immunity and Cardiovascular Disease, School of Medicine, Dentistry and Health, University of Sheffield, Sheffield, UK; 2 School of Life and Health Sciences, Aston University, Birmingham, Birmingham, UK; 3 Department of Respiratory Medicine, National Heart and Lung Institute, Imperial College London, London, UK; 4 School of Medicine, Institute of Infection and Immunity, Cardiff University, Cardiff, UK; 5 Department of Biomedical Sciences, Faculty of Science, University of Sheffield, Sheffield, UK; 6 Department of Pathology, University of Cambridge, Cambridge, UK; 7 School of Biosciences, Cardiff University, Cardiff, UK

**Keywords:** Inflammation, Biologics, Biomaterials, Viral infection

## Abstract

Human rhinovirus (HRV) infections are major contributors to the healthcare burden associated with acute exacerbations of chronic airway disease, such as chronic obstructive pulmonary disease and asthma. Cellular responses to HRV are mediated through pattern recognition receptors that may in part signal from membrane microdomains. We previously found Toll-like receptor signaling is reduced, by targeting membrane microdomains with a specific liposomal phosphatidylserine species, 1-stearoyl-2-arachidonoyl-*sn*-glycero-3-phospho-L-serine (SAPS). Here we explored the ability of this approach to target a clinically important pathogen. We determined the biochemical and biophysical properties and stability of SAPS liposomes and studied their ability to modulate rhinovirus-induced inflammation, measured by cytokine production, and rhinovirus replication in both immortalized and normal primary bronchial epithelial cells. SAPS liposomes rapidly partitioned throughout the plasma membrane and internal cellular membranes of epithelial cells. Uptake of liposomes did not cause cell death, but was associated with markedly reduced inflammatory responses to rhinovirus, at the expense of only modest non-significant increases in viral replication, and without impairment of interferon receptor signaling. Thus using liposomes of phosphatidylserine to target membrane microdomains is a feasible mechanism for modulating rhinovirus-induced signaling, and potentially a prototypic new therapy for viral-mediated inflammation.

**Supplementary information:**

The online version of this article (doi:10.1038/mi.2015.137) contains supplementary material, which is available to authorized users.

## Introduction

Human rhinoviruses (HRVs) typically cause mild infections of the upper respiratory tract. More recently, it has become evident that HRV infections trigger a high proportion of acute exacerbations in airway diseases such as asthma and chronic obstructive pulmonary disease (COPD),^1^ and have also been implicated in other respiratory disorders such as pneumonia.^2^ Despite the frequency of these infections, there are currently no effective specific therapies for HRV-induced inflammation, and the multiple serotypes of HRV preclude successful vaccination.

HRVs are nonenveloped, positive sense single-stranded RNA (ssRNA) members of the *Picornaviridae* family of viruses. Presently, there are more than 150 identified HRV serotypes divided into distinct groups; HRV-A, HRV-B, and HRV-C, according to their phylogenetic similarity.^3^ HRV-A and -B attach and gain entry to cells via the receptors intracellular adhesion molecule 1 (ICAM-1) and low-density-lipoprotein receptor, while the receptor for HRV-C serotypes has yet to be identified.^4, 5, 6^ Receptor-mediated internalization of HRV occurs via membrane microdomains enriched in cholesterol or ceramide,^7^ and evidence suggests that early signaling responses to HRV infection results in activation of Src and Akt which may also occur from these regions.^8^ Membrane microdomains such as lipid rafts are highly dynamic subdomains of the cell membrane, which act to facilitate protein–lipid and protein–protein interactions and signaling.^9^ These domains have important roles in promoting signaling events by many immune receptors, including the pattern recognition receptors (PRRs), Toll-like receptors (TLRs).^10^ Infected airway epithelial cells detect and respond to HRV via TLRs and the RNA helicases retinoic acid-inducible gene I (RIG-I) and melanoma differentiation-associated gene 5 (MDA5), activating signaling pathways that cause the generation of pro-inflammatory cytokines and type I interferons (IFNs). Cell surface recognition of viral capsid proteins occurs by TLR2,^11^ while receptors such as TLR7/8 and RIG-I/ MDA5 localize to the endosome and cytosol, respectively, and detect single stranded (TLR7/8) or double stranded viral RNA forms.^12^ Remodeling of membrane microdomain structure through the addition or removal of nonsterol phospholipids has been associated with effects on signaling,^13^ and we have previously demonstrated that TLR signaling is inhibited by liposomes consisting of a specific phosphatidylserine (PS) species, 1-stearoyl-2-arachidonoyl-*sn*-glycero-3-phospho-L-serine, which disrupted the membrane-microdomain-dependent association of TLR4 with its co-receptor CD14.^14^

Herein, we examined whether therapeutic targeting of viral-induced TLR-dependent inflammation is feasible using SAPS liposomes. The roles of membrane microdomains have been traditionally studied using detergent extraction and cholesterol depletion methods.^9^ However, results obtained using these techniques are hard to interpret with precision, as detergent extraction is highly dependent on experimental conditions, and cholesterol depletion can result in global cellular functional disruption.^9^ Recent work has also demonstrated that cholesterol trafficking is required for effective picornavirus replication,^15^ therefore depleting cholesterol to modulate membrane microdomain function may have important consequences on viral replication by other routes. In this study, we utilized SAPS to explore the roles of membrane microdomains in viral signaling processes without globally perturbing membrane structure. We examined the stability of SAPS, created stable uniform-sized liposomes, and determined their effects on HRV infection of airway epithelial cells. Confocal studies revealed that SAPS was rapidly internalized and associated with sites of importance for HRV-induced signaling. SAPS notably impaired HRV-induced cytokine responses, and also reduced HRV-induced IFN-β production and signal transducers and activators of transcription (STAT)-1 phosphorylation. Viral replication rates were not significantly altered, and only modest increases in viral particle production were observed. Thus SAPS is a promising new tool to study the role of membrane microdomains in viral infections, and has the potential for future development as a novel therapeutic strategy in the prevention of airway inflammation within chronic pulmonary diseases such as asthma and COPD.

## Results

### Characterization and intracellular distribution of SAPS liposomes

We initially investigated the stability of experimental 1-stearoyl-2-arachidonoyl-*sn*-glycero-3-phospho-L-serine (SAPS) liposomes and control 1-palmitoyl-2-arachidonoyl-*sn*-glycero-3-phosphocholine (PAPC) liposomes prepared in phosphate buffered saline (PBS) according to the lipid hydration method.^16^ Blending lipids with differing hydrocarbon lengths and saturation can alter physical properties of liposomes. We therefore additionally determined whether incorporation of 25% 1,2-distearoyl-*sn*-glycero-3-phosphocholine (DSPC) to SAPS would affect liposomal stability and activity.^17^ All liposomes were of a potentially respirable mean sub-micrometer size, though control PAPC liposomes were larger than SAPS and SAPS+DSPC liposomes, most likely reflecting structural and molecular differences in the polar head group and fatty acid chains between the molecules (Supplementary Table S1 online). Mean liposome sizes were 203.6±48.0 nm for SAPS and 629.6±90.7 nm for PAPC. Liposomes retained their size distribution up to the latest tested point (28 days) in storage at 4 °C (Supplementary Figure S1). Polydispersity values were low (Supplementary Table S1), indicating that formulations were homogenous in nature. Differential scanning colorimetry was exploited to investigate the transition temperatures of liposomes. SAPS, SAPS+DSPC, and PAPC exhibited a single endothermic response with an onset temperature of 17.48 °C, 18.19 °C, and 16.67 °C, respectively (Supplementary Figure S2) consistent with bilayer fluidity above these temperatures.^18^ Although DSPC is commonly used to increase liposome stability, addition of DSPC was not required for liposomal stability judged by sizing, nor did it alter biological actions of SAPS (data not shown), and hence was not routinely added to liposomes. No traces of SAPS oxidation products were observed in a weekly mass spectrometric analysis over 4 weeks (Supplementary Figure S3).

**Figure 1:**
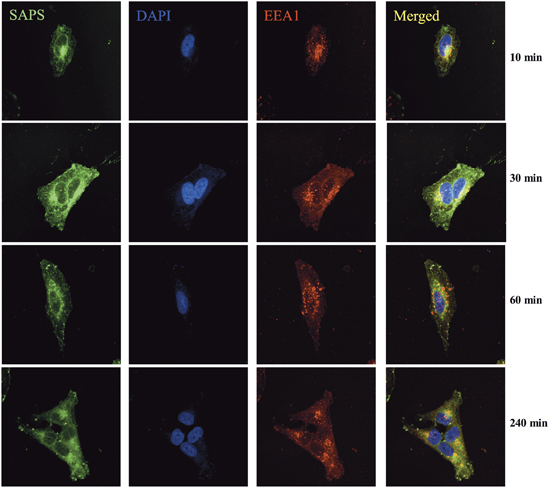
Endosomal localized 1-stearoyl-2-arachidonoyl-*sn*-glycero-3-phospho-L-serine (SAPS). BEAS-2B cells were incubated with TopFluor-SAPS (100 μg ml^−1^) for indicated time periods. Intracellular distribution of SAPS (green) partially colocalized within endosomes (EEA1/red). Images were collected using a Nikon confocal microscope and are representative of three independent experiments. The merged image shows overlay of areas positive for SAPS and endosome. PowerPoint slide

**Figure 2:**
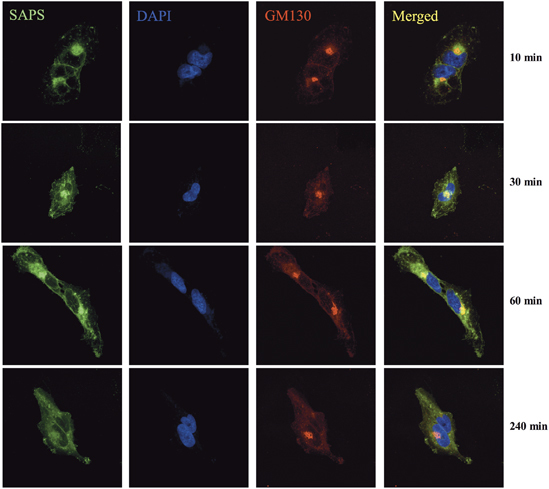
Golgi localized 1-stearoyl-2-arachidonoyl-*sn*-glycero-3-phospho-L-serine (SAPS). BEAS-2B cells were incubated with TopFluor-SAPS (100 μg ml^−1^) for indicated time periods. Intracellular distribution of SAPS (green) partially colocalized with the golgi (GM130/red). Images were collected using a Nikon confocal microscope and are representative of three independent experiments. The merged image shows overlay of areas positive for SAPS and the Golgi. PowerPoint slide

HRV infection initiates intracellular signaling events through the recognition of viral RNA structures by the endosomal located pattern recognition receptor, TLR3.^12^ To determine whether SAPS targets sites of importance for viral signaling, we examined the intracellular distribution of fluorescent SAPS (TopFluor-SAPS) within the bronchial epithelial cell line BEAS-2B.^19^ Strikingly, TopFluor-SAPS rapidly distributed throughout the cell within 10 min, quickly reaching endosomal compartments from which TLR3 signaling is initiated (Figure 1). Furthermore, diffusion to Golgi membranes was also observed (Figure 2).

### SAPS inhibits cytokine production induced by HRV infection

**Figure 3:**
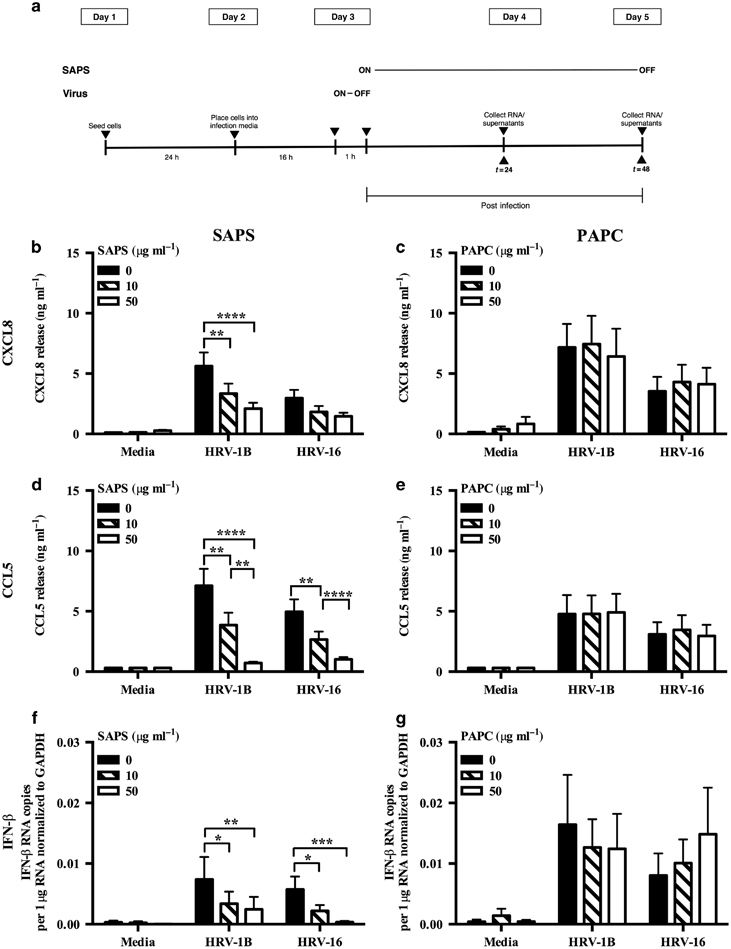
1-Stearoyl-2-arachidonoyl-*sn*-glycero-3-phospho-L-serine (SAPS) modulates human rhinovirus (HRV)-induced cytokine production. (**a**) Experimental design. BEAS-2B cells infected with HRV (MOI 3/TCID_50_ per ml 1 × 10^7^) were treated with SAPS or 1-palmitoyl-2-arachidonoyl-*sn*-glycero-3-phosphocholine (PAPC) at the doses indicated. After 24 h, CXCL8 (**b**, **c**) or CCL5 (**d**, **e**) release were measured, and IFN-β mRNA expression quantified and presented as total IFN-β mRNA copies per 1 μg RNA normalized to GAPDH expression (**f**, **g**). Data shown are mean±s.e.m. (*n*=11 for **b**, *n*=9 for **c**, and *n*=6 for **d**, **f,** and **g**). Significant differences are indicated by *P<0.05, **P<0.01 ***P<0.001, and ****P<0.0001. PowerPoint slide

**Figure 4:**
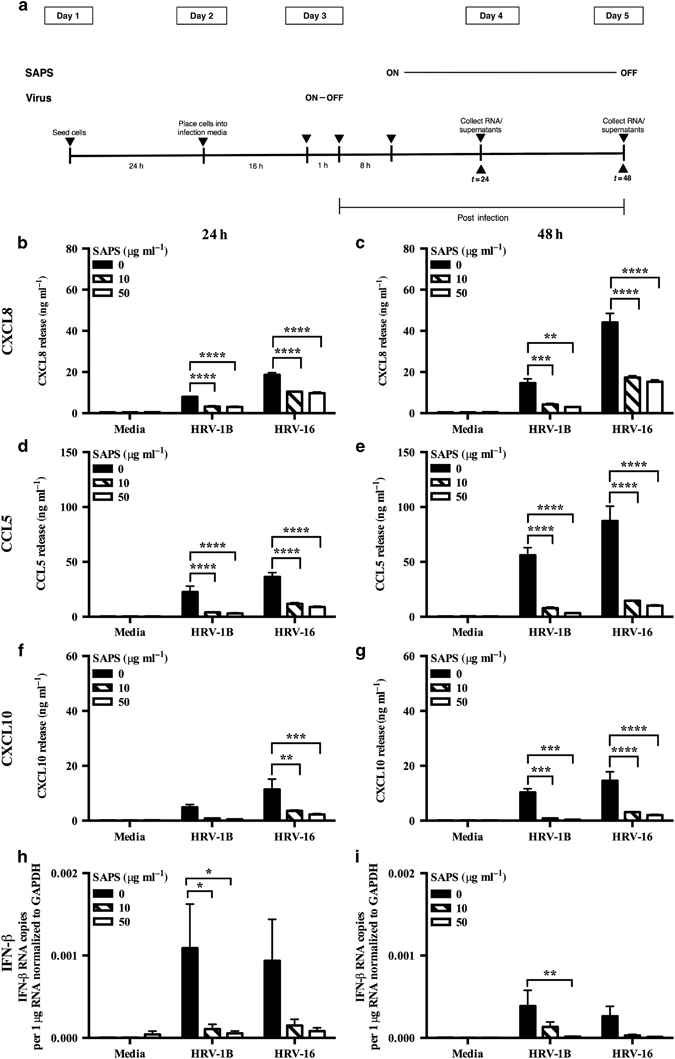
1-Stearoyl-2-arachidonoyl-*sn*-glycero-3-phospho-L-serine (SAPS) modulates established human rhinovirus (HRV)-induced cytokine production. (**a**) Experimental design. BEAS-2B cells infected with HRV (MOI 3/TCID_50_ per ml 1 × 10^7^) were treated with SAPS at 8 h post-viral internalization at the doses indicated. After 24 h (**b**, **d**, **f**, **h**) or 48 h (**c, e, g, i**), CXCL8 (**b, c**), CCL5 (**d, e**), and CXCL10 (**f, g**) release were measured, and IFN-β mRNA expression quantified and presented as total IFN-β mRNA copies per 1 μg RNA normalized to GAPDH expression (**h, i**). Data shown are mean±s.e.m. (*n*=3). Significant differences are indicated by *P<0.05, **P<0.01, ***P<0.001, and ****P<0.0001. PowerPoint slide

We have previously found that SAPS inhibited inflammatory responses to several TLR agonists through disruption of membrane microdomains.^14^ Whether this translates into biologically useful inhibition of signaling during pathogen-mediated inflammation is unknown. We therefore determined if SAPS would alter the roles of membrane microdomains in cellular function and control of HRV. SAPS was either added at 4 h prior to viral infection of cells to study effects of SAPS on cell attachment and internalization, or at several time points post viral infection to study the effects of SAPS on early signaling events. We examined CXCL8 and CCL5 production, downstream targets of nuclear factor-kappa B (NF-κB), and interferon regulatory factor 3 (IRF-3) activation. We also examined the production of CXCL10, a proinflammatory chemokine with a potential role in viral-induced exacerbations.^20^ Viral titers and SAPS concentrations were as previously described and resulted in no notable cytopathic effects.^14, 21^ First, we assessed the kinetics of proinflammatory chemokine production and found that CXCL10 production could be detected from as early as 8 h post HRV infection, with CXCL8 and CCL5 production predominately detected from 16 h onward (Supplementary Figure S4). SAPS reduced CXCL10, (Supplementary Figure S4e,f), CXCL8 (Figure 3b; Supplementary Figure S4a,b), and CCL5 (Figure 3d; Supplementary Figure S4c,d) production from HRV-infected epithelial cells when added at 1 h post viral challenge of the cells. To evaluate the efficacy of SAPS treatment following the establishment of viral infection, SAPS was also added 4 and 8 h after viral challenge. The addition of SAPS at 4 and 8 h post viral infection reduced cytokine production equally well (data shown for 8 h post viral infection, Figure 4b–g), demonstrating that, even after HRV infection has begun to establish, SAPS addition can still act to reduce inflammatory mediator production from bronchial epithelial cells. The actions of SAPS were most notable upon CCL5 and CXCL10 release. Both CCL5 and CXCL10 are considered interferon-stimulated genes, and production is induced following activation of an IFN-β-dependent autocrine loop. In keeping with these data, HRV infection caused marked induction of IFN-β mRNA relative to media controls, which was significantly inhibited by SAPS (Figure 3f; Figure 4h,i; and Supplementary Figure S4g,h). Type III interferons, consisting of the three IFN-λ subtypes are co-produced with IFN-β.^22^ Similarly to what was observed with IFN-β induction, SAPS treatment also significantly inhibited IFN-λ1 and IFN-λ2 induction (data not shown). In contrast, CXCL8, CCL5, and IFN-β production were not significantly altered following treatment with PAPC (Figure 3c,e,g). We next investigated the actions of SAPS on virus internalization and infection. Cells were pretreated with SAPS for 4 h then subsequently infected with HRV. In the setting of treatment before viral infection, SAPS treatment did not significantly alter HRV-1B-induced CXCL8 (Supplementary Figure S5a,b) and CCL5 production (Supplementary Figure S5c,d). However, the highest concentration of SAPS tested did modestly reduce HRV-16-induced CXCL8 and CCL5 production.

**Figure 5:**
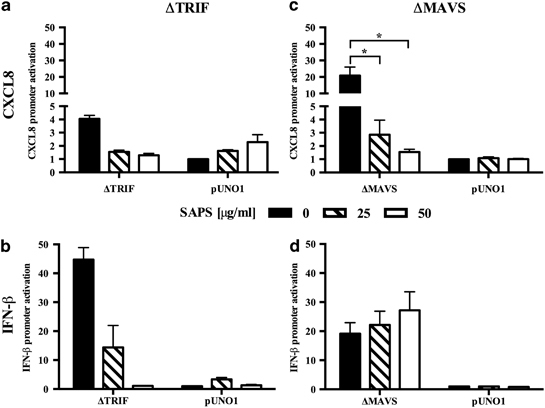
1-Stearoyl-2-arachidonoyl-*sn*-glycero-3-phospho-L-serine (SAPS) modulates TRIF- and MAVS-dependent activation of the CXCL8 and IFN-β promoters. BEAS-2B cells were transfected with plasmids encoding ΔTRIF and MAVS. After 6 h, transfected cells were treated with the indicated SAPS concentrations and cell lysates were generated at 24 h. ΔTRIF (**a, b**) or MAVS (**c, d**) induced CXCL8 (**a, c**), and IFN-β (**b, d**) reporter activation relative to empty vector control pUNO1 was measured. Data shown are mean±s.e.m. (*n*=2 for **a** and **b**, and *n*=5 for **c** and **d**). Reporter data are presented as fold induction vs. control. Significant differences are indicated by *P<0.05. PowerPoint slide

The TLR3 signaling adapter TIR domain-containing adapter inducing IFN-β (TRIF) associates with membrane microdomains in the context of TLR4.^23^ However, little is known about the dependence of TLR3 on membrane microdomain signaling. To verify that SAPS treatment inhibited TLR3 signaling directly, we investigated the effect of SAPS liposome treatment on TLR3/TRIF-dependent signaling by transfecting epithelial cells with plasmids expressing a constitutively active form of TRIF (ΔTRIF) and reporters for CXCL8 and IFN-β.^12^ ΔTRIF induced CXCL8 and IFN-β promoter activation, and this was reduced by SAPS (Figure 5). The actions of SAPS were most notable on IFN-β promoter activation (Figure 5b). HRV-induced TLR3 activation subsequently leads to the upregulation of the RNA helicases RIG-I and MDA5.^12^ Activation of inflammatory processes by these RNA helicases is mediated by the adapter protein mitochondrial antiviral signaling (MAVS) (also known as CARDIF, IPS-1, and VISA) which resides at the mitochondrial outer membrane.^24^ The mitochondrial membrane may also be organized to include specific microdomains.^25^ Overexpression of full-length MAVS was performed in BEAS-2B epithelial cells.^24^ Activation of the CXCL8 promoter by MAVS, but not activation of the IFN-β promoter, was inhibited by SAPS (Figure 5c,d).

### Modulation of IFN-β expression by SAPS is not via direct effects on IFN signaling pathways

**Figure 6:**
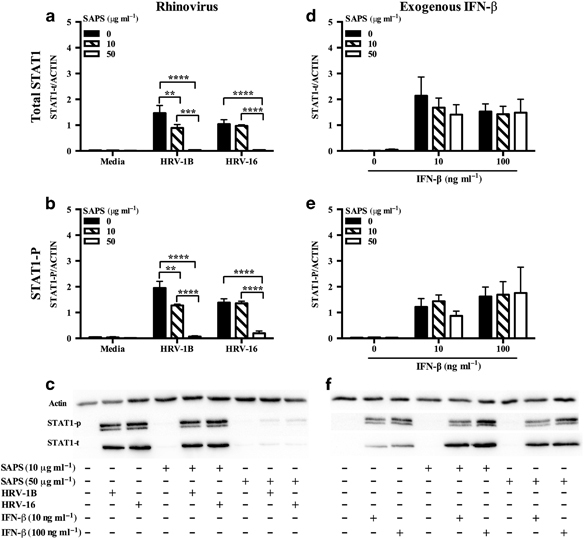
The actions of 1-stearoyl-2-arachidonoyl-*sn*-glycero-3-phospho-L-serine (SAPS) are not through modulation of the Type I IFN receptor (IFNAR). BEAS-2B cells were infected with human rhinovirus (HRV) (MOI 3) (**a-c**), then treated with SAPS; or pre-treated with SAPS for 1 h prior to the addition of IFN-β (10 and 100 ng ml^−1^) (**d-f**). Whole-cell lysates were collected at 24 h. A representative blot of three (**c**) or four (**f**) independent experiments is shown. Data shown are mean±s.e.m. (*n*=3 for **a** and **b**, and *n*=4 for **d** and **e**). Significant differences are indicated by **P<0.01, ***P<0.001, and ****P<0.0001. PowerPoint slide

Early production of IFNs activates an autocrine loop dependent on Type I IFN receptor (IFNAR) signaling resulting in STAT1 activation. IFNAR signaling may be partly dependent on membrane microdomain function.^26^ To determine if some actions of SAPS might be via inhibition of IFNAR, we examined if SAPS modified HRV-induced STAT1 phosphorylation. Induction of total STAT1 protein and STAT1 phosphorylation was observed in HRV-infected cells at 24 h, and this was reduced following SAPS treatment (Figure 6a–c). The inhibitory actions of SAPS were greater at the highest concentration tested (50 μg ml^−1^). High concentrations of PAPC also modestly reduced total STAT1 and STAT1 phosphorylation (Supplementary Figure S6a–c). To ascertain whether SAPS was acting prior to, or downstream of IFNAR, we examined whether addition of exogenous IFN-β would recover total STAT1 protein levels and STAT1 phosphorylation in the presence of SAPS. IFN-β stimulation rescued the production of total STAT1 protein and STAT1 phosphorylation in the presence of SAPS at both concentrations tested (Figure 6d–f).

### SAPS has only modest effects on viral replication

**Figure 7:**
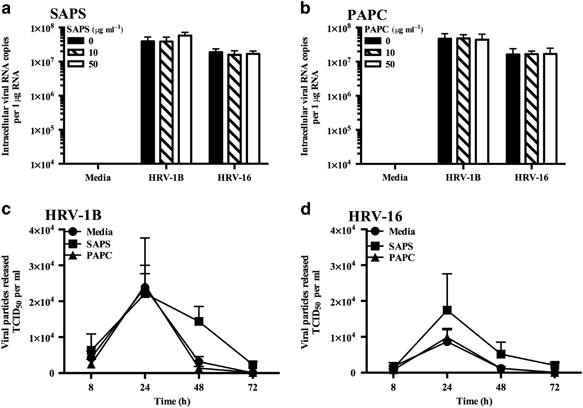
1-Stearoyl-2-arachidonoyl-*sn*-glycero-3-phospho-L-serine (SAPS) treatment has modest effects on viral replication. BEAS-2B cells were infected with human rhinovirus (HRV) (MOI 3/TCID_50_ per ml 1 × 10^7^), and treated with (**a**) SAPS or (**b**) 1-palmitoyl-2-arachidonoyl-*sn*-glycero-3-phosphocholine (PAPC). Intracellular viral RNA expression was quantified at 24 h, with data presented as the total intracellular viral RNA copies per 1 μg RNA. Viral particle release into the supernatant was quantified at the indicated time periods following liposome treatment (50 μg ml^−1^) (**c, d**). Data shown are mean±s.e.m. (*n*=6 for **a** and *n*=4 for **b**–**d**). Significant differences are indicated by *P<0.05 and **P<0.01. PowerPoint slide

**Figure 8:**
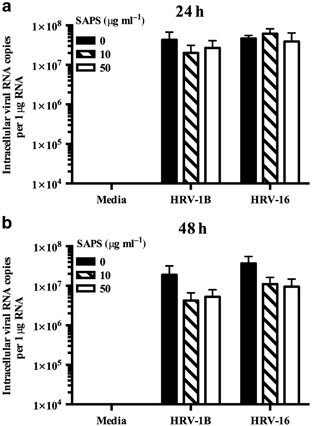
1-Stearoyl-2-arachidonoyl-*sn*-glycero-3-phospho-L-serine (SAPS) addition 8 h post virus internalization does not effect viral replication. BEAS-2B cells were infected with human rhinovirus (HRV) (MOI 3/TCID_50_ per ml 1 × 10^7^), and treated with SAPS at 8 h post virus internalization. Intracellular viral RNA expression was quantified at 24 h (**a**) and 48 h (**b**) with data presented as the total intracellular viral RNA copies per 1 μg RNA. Data shown are mean±s.e.m. (*n=*3). PowerPoint slide

We observed significant reductions in IFN generation and signaling in epithelial cells treated with SAPS, and therefore determined whether this would result in increased viral replication. HRV-infected BEAS-2B cells were quantified for intracellular viral RNA levels by quantitative PCR (qPCR). No significant effects on viral replication were observed following treatment with SAPS or control PAPC when added at 1 h post viral challenge (Figure 7a,b; Supplementary Figure S7). The release of infective HRV-1B from BEAS-2B cells did not significantly differ between control, SAPS-, or PAPC-treated cells, although modest non-significant increases following SAPS treatment were observed at 48 h (Figure 7c). Release of HRV-16 was also modestly elevated following SAPS treatment at 24 and 48 h, again this did not reach statistical significance (Figure 7d). The addition of SAPS 4 h post viral infection did not notably effect viral replication (data not shown), although moderate increases in HRV-16 replication were detected at SAPS concentrations of 50 μg ml^−1^ at 24 h only. Replication did not differ significantly between control or SAPS-treated cells, when SAPS was added at 8 h post viral infection, although moderate non-significant decreases in viral replication were detected at 48 h following SAPS treatment (Figure 8b).

### PRR is not altered by SAPS

Activation of PRRs following HRV infection results in the downstream activation of transcription factors such as IRF-3. Given the reduction in IFN production consequent upon SAPS treatment of virally infected cells, we determined whether there was also suppression of PRR and IRF expression. HRV infection induced TLR3, RIG-I, MDA5, IRF-1, and IRF-7 relative to media controls, and this was not inhibited by SAPS treatment (Supplementary Figures S8 and S9). Expression of IRF-3 and MAVS were unaltered in the presence or absence of HRV or SAPS.

### SAPS regulated HRV-induced inflammatory responses in normal and diseased primary bronchial epithelial cells

**Figure 9:**
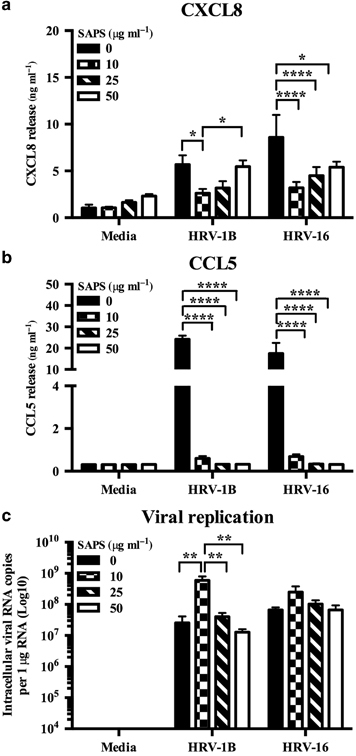
1-Stearoyl-2-arachidonoyl-*sn*-glycero-3-phospho-L-serine (SAPS) selectively modulates human rhinovirus (HRV)-induced cytokine production from primary bronchial epithelial cells (PBECs). PBECs were infected with HRV (MOI 3/TCID_50_ per ml 1 × 10^7^), followed by SAPS (**a**–**c**) treatment at the doses indicated. After 48 h, CXCL8 (**a**) and CCL5 (**b**) release were measured, and intracellular viral RNA expression was quantified (**c**) with data presented as the total intracellular viral RNA copies per 1 μg RNA. Data shown are mean±s.e.m. (*n*=4) with each replicate performed on independent donors. Significant differences are indicated by *P<0.05, **P<0.01, *** and ****P<0.0001. PowerPoint slide

**Figure 10:**
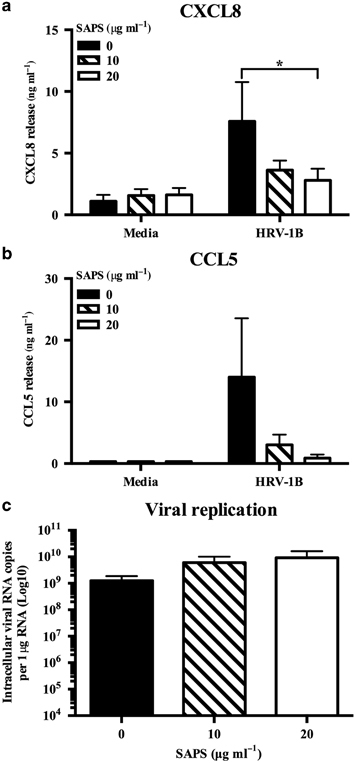
1-Stearoyl-2-arachidonoyl-*sn*-glycero-3-phospho-L-serine (SAPS) modulates human rhinovirus (HRV)-induced cytokine production from primary bronchial epithelial cells (PBECs) isolated from chronic obstructive pulmonary disease (COPD) patients. PBECs isolated from people with COPD were infected with HRV (MOI 0.1/TCID_50_ per ml 1.3 × 10^5^), followed by SAPS (**a**–**c**) treatment at the doses indicated. After 48 h, CXCL8 (**a**) and CCL5 (**b**) release were measured, and intracellular viral RNA expression was quantified with data presented as the total intracellular viral RNA copies per 1 μg RNA. Data shown are mean±s.e.m. (*n*=3) with each replicate performed on independent donors. Significant differences are indicated by *P<0.05. PowerPoint slide

We next examined the actions of SAPS on HRV infection of human primary bronchial epithelial cells (PBECs). Compared with BEAS-2B cells, SAPS exerted less inhibition of CXCL8 but very marked inhibition of CCL5 production (Figure 9). No significant effects on viral replication were observed when SAPS was present at concentrations of 25 or 50 μg ml^−1^ (Figure 9c), but moderate increases in viral replication were detected at SAPS concentrations of 10 μg ml^−1^. HRV infections are important triggers of acute episodes within chronic diseases such as asthma and COPD. Pro-inflammatory mediators such as CXCL8 and CCL5 generated during infection potentially perpetuate underlying chronic inflammation,^27, 28^ while pre-existing allergic inflammation may be enhanced by HRV infection.^29^ We hypothesized that SAPS would also be effective in disease, and examined the actions of SAPS on HRV infection of human PBECs obtained from people with COPD. Epithelial cells from people with COPD were more susceptible to HRV infection and SAPS treatment. Accordingly, cells were infected with HRV-1B at a reduced viral titer (1.3 × 10^5^ TCID_50_ per ml) and treated with SAPS concentrations (10 and 20 μg ml^−1^) that resulted in cytopathic effects that were comparable to those observed in normal PBECs. SAPS reduced CXCL8 (Figure 10a) and CCL5 (Figure 10b) production from HRV-1B-infected epithelial cells, with the actions of SAPS most notable for CCL5 release. No significant effects on viral replication were observed (Figure 10c). Human primary airway epithelial cells cultured at air–liquid interface (ALI) are regarded to better represent the characteristics of normal respiratory epithelium *in vivo*.^30, 31^ We therefore examined the uptake of TopFluor-SAPS within PBECs differentiated at ALI. PBECs cultured at ALI expressed markers of basal cell differentiation such as ciliated bronchial epithelium 1 (CBE1) and Mucin 5B (MUC5B) as shown by reverse-transcriptase PCR (RT-PCR) (Supplementary Figure S10a), and β-Tubulin as shown by immunofluorescence confocal microscopy (Supplementary Figure S10b) confirming that the primary cells had effectively differentiated into a mucociliary phenotype.^32^ TopFluor-SAPS internalized into the cell as quickly as 30 min post application (data not shown). Within 180 min, most of the TopFluor-SAPS diffused to intracellular regions as determined by confocal Z-stack imaging (Supplementary Figure S11).

## Discussion

Respiratory viruses, such as HRVs are important drivers of acute exacerbations of chronic pulmonary diseases. Our previous work has established that inhibition of TLR signaling by a phosphatidylserine species (SAPS) occurs through a mechanism that is dependent upon the disruption of TLR signaling complexes within membrane microdomains.^14^ Various lines of evidence show that membrane microdomains are sites of attachment and cell entry for HRV, and that early cellular inflammatory signaling is mediated from such domains.^7, 8, 33^ Furthermore, viral cell entry requires acidification and maturation of endosomes,^34^ TLR3 signals from endosomes and is implicated in recognition of responses to HRV,^11, 12^ and membrane reorganization of the cell is required for viral replication.^35^ These studies raise the possibility that SAPS could potentially alter HRV infectivity, inflammatory signaling, and replication by targeting membrane microdomain function, and as such be useful as a treatment for HRV infection.

In this study, we reveal that SAPS liposomes can regulate innate immune responses activated by HRV infection. The addition of SAPS liposomes at various times following viral infection suppressed the release of the proinflammatory cytokines, CXCL8 and CXCL10, as well as the interferon-stimulated gene CCL5/RANTES from epithelial cell lines and normal and diseased primary human bronchial epithelial cells. In keeping with reductions in CCL5 production, viral-induced type I IFN generation was also reduced, with the highest tested concentration of SAPS also inhibiting STAT1 induction and phosphorylation. Although reductions in IFN production were observed, this did not appear to affect viral replication, as in most cases viral replication was unimpeded by liposome treatment in the BEAS-2B epithelial cell line and in human PBECs. Furthermore, similar replication characteristics were observed regardless of whether cells were from normal individuals or people with COPD. This suggests that the potential mechanism of action of SAPS is not via direct interference of virus attachment and internalization, and SAPS is therefore not preventing subsequent cycles of viral infection. Indeed SAPS treatment before viral challenge with removal of liposomes immediately before viral infection further validates this conclusion, as it does not substantially diminish HRV-induced CXCL8 and CCL5 induction. In addition, although studies indicate that minor group and major group rhinoviruses enter cells through distinguishable mechanisms, and uncoat and infect from distinct endosomal populations,^33, 36, 37^ no notable differences were observed between the two HRV serotypes studied here. Another possible mechanism of action is that part of the reduction in CCL5 production is due to the direct disruption of type I IFN autocrine actions by SAPS, given that the downstream effectors of the IFNAR, JAK1, and STAT1 associate with caveolar regions; a specialized sub-domain of membrane lipid rafts.^38^ However, our *in vitro* studies rule out this possibilty as exogenous IFN-β induced STAT1 in the presence of SAPS. Furthermore, our data also demonstrate that antiviral defense through the activation of antiviral effectors is still occurring, as inducible and constitutive PRR and IRF expression was not inhibited by SAPS, and inhibition of IFN production did not result in enhanced viral replication. It should be noted that variations on the dependence of IFN to induce interferon-stimulated genes have been reported.^39, 40, 41^ Our data provide strong evidence that IFNAR is for the most part not dependent on membrane microdomains and that reductions in CCL5 production are likely a result of SAPS acting further upstream to diminish IFN production or p65 activity.^42^

The PRR, TLR3 is crucial for mediating responses to rhinovirus,^12^ and is localized to the endosome. To determine if disruption of TLR3 signaling by direct actions of SAPS was a feasible mechanism of inhibition, we studied the intracellular trafficking of SAPS. Fluorescent SAPS partitioned rapidly through both basal and differentiated epithelial cells, appearing to quickly reach intracellular regions. Such a rapid uptake and distribution of phosphatidylserine species through the cell has been observed in recent work from another group,^43^ and is in keeping with older data obtained in fibroblasts.^44^ The mechanisms of uptake of SAPS have yet to be characterized, though appear to be an active phospholipid-specific pathway.^44^ Further supporting potential direct targeting of the TLR3 signaling pathway, we also observed a reduction in TRIF-dependent signaling following SAPS treatment. When HRV infects epithelial cells, TLR3 signaling is followed by activation of the RNA helicases, RIG-I, and MDA5. Membrane microdomains have been detected within endosomal and mitochondrial membranes, the sites at which TLR3/RNA helicase signaling adapters dock respectively^25, 45^ To determine whether SAPS targeted RNA helicase signaling pathways, we also studied effects on MAVS-dependent signaling. Interestingly, SAPS reduced MAVS-dependent activation of CXCL8, without impairing IFN-β activation. Very recent work has shown that binding of eukaryotic elongation factor 1 Bγ (eEF1Bγ) to MAVS at the mitochondrial membrane positively regulates the activation of the transcription factor NF-κB and pro-inflammatory cytokines such as CXCL8.^46^ One potential explanation for our observations is that SAPS is acting to modulate signaling at the mitochondrial membrane, thereby preventing recruitment of eEF1Bγ. In addition, MAVS has been shown to reside on peroxisomes as well as at the mitochondrial membrane, with variations in antiviral signaling from each subcellular compartment.^40^ Our data suggest the possibility that activation of IFN pathways by MAVS is dependent upon downstream protein–protein interactions that are either in domains not targeted by SAPS, or are independent of microdomain function. Furthermore, it is feasible that SAPS only selectively targets particular components of MAVS signaling. As our data above show that MAVS activation of IFN is not inhibited by SAPS, these data imply that the majority of the induction of IFN by HRV over the time course studied here is mediated through TLR3 signaling. Consistent with previous studies,^14^ we again confirm that SAPS does not result in a global disruption of receptor-mediated signaling, with anti-viral responses selectively suppressed by SAPS. Furthermore, preservation of the induction of downstream antiviral PRRs in response to HRV infection was observed, supporting our view that potential targets of SAPS are further upstream.

The use of SAPS as a potential anti-inflammatory has several potential advantages. First, it is a completely naturally occurring phospholipid,^14^ which has so far proved non-toxic in bacterial and viral animal challenge studies (unpublished observations). Furthermore, as it is naturally occurring, it will be processed through normal physiological mechanisms,^47^ and is unlikely to have adverse drug interactions. Second, our current *in vitro* studies demonstrate that all liposomes prepared throughout the study showed size characteristics within the submicrometer respirable range (<5 μm)^48^ thus supporting a potential therapeutic application for delivery to the respiratory tract in a nebulized form. Other studies indicate that liposomal size stability has important effects on the biological activity of the liposome formulations, with oxidation during storage a significant influencing factor.^49, 50^ Our liposomes were stable for more than 1 month’s storage, and analyses revealed no notable traces of SAPS oxidation. Moreover, although liposomal characteristics can be significantly affected by the choice of lipid species, with vesicle size notably influencing biodistribution,^51^ the addition of the lipid DSPC to SAPS was of no added benefit, suggesting that SAPS liposomes alone would be stable enough to be made in advance of any clinical use. Third, the stability and simplicity of SAPS also make it an appealing candidate therapeutic in terms of cost. Last, given that antiviral properties have been demonstrated for other lipid species,^52, 53^ it is feasible that delivery of SAPS in combination with other lipid components may enhance its potency as both an anti-inflammatory and anti-viral agent. Although our research currently focuses on the use of empty liposomes to modulate airway inflammation, the packaging of these liposomes to contain IFN is also a possibility. Especially as our data demonstrates that exogenous IFN can still activate downstream signaling via the IFNAR in the presence of SAPS. Given that our data demonstrates a reduced ability to produce interferon, albeit without adverse consequences on viral replication, this may be advisable. Several studies have implicated defective IFN induction as a potential mechanism for driving virus-induced asthma exacerbations,^54, 55^ although, others have failed to reproduce these results.^56, 57^ We could conceivably supplement the anti-inflammatory actions of SAPS with IFN. Thus activating anti-viral signaling pathways that would limit viral replication, and subsequent spread of viral infection. Such considerations are the potential subject of future experiments.

Although SAPS has appreciable therapeutic potential, there is reason for caution. Although key experiments were conducted in undifferentiated normal and diseased human PBECs, a large proportion of the data was generated using the immortalized epithelial cell line, BEAS-2B in an acute lung model. Thus the results may not fully resemble what may occur *in vivo* within chronic lung diseases such as asthma and COPD, and the wider implications of using such liposomes in more complex models are unclear. Furthermore, despite initial characterization studies indicating potential liposome delivery to the lung, the distribution and deposition of these liposomes *in vivo* has yet to be examined. Future studies may involve studying the action of SAPS in a relevant chronic inflammation model that reproduces many of the features of asthma or COPD.^58, 59^

Nevertheless, our data show that SAPS liposomes have the ability to modulate HRV-induced inflammatory signaling *in vitro*, without significant effects on viral replication in both normal and diseased epithelial cells. We have previously also demonstrated that SAPS can efficiently disrupt communicative dialog between cocultures of tissue cells and leukocytes in response to TLR agonists *in vitro*.^14^ Thus while a continued understanding of the effects of SAPS liposomes on other epithelial cell functions and on the development of adaptive immunity is clearly essential for the potential development of SAPS as an anti-inflammatory therapy to regulate airway inflammation, SAPS liposomes represent an exciting therapeutic prospect and a new tool to explore membrane microdomain roles in regulating viral signaling processes.

## Methods

**Cell culture.** BEAS-2B epithelial and HeLa OHIO cell lines were obtained from ATCC (LGC Standards, Teddington, UK) and ECACC (Sigma-Aldrich, Dorset, UK), respectively, and maintained as previously described.^21^ PBECs isolated from healthy humans were purchased from Promocell (Heidelberg, Germany) and PBECs isolated from three patients with COPD were purchased from Lonza (Basel, Switzerland). Cells were maintained as previously described.^60^

**Differentiation of PBECs.** Normal PBECs (Promocell) were initially cultured in BEGM medium (Promocell) as previously described.^60^ Cells were obtained from one individual donor, seeded into T25 flasks, and subcultured with Trypsin-EDTA. At confluency, PBECs were subcultured with Trypsin-EDTA and re-suspended in ALI media consisting of 50% DMEM (Invitrogen, Paisley, UK), 50% BEGM plus all pack supplements except retinoic acid (Promocell), retinoic acid (100 nM) (Sigma-Aldrich), and BSA (1.5 mg ml^−1^) (Sigma-Aldrich). Cultures were seeded at 0.5 × 10^6^ cells per ml onto the apical surface of Collagen I (Becton Dickinson, Oxford, UK) coated transwell PET inserts with 0.4 μm pore size (FALCON; Becton Dickinson) and placed in 24-well flat-bottomed plates with 0.75 ml of ALI media in the basal and 0.25 ml of ALI media in the upper compartments. Media in both compartments was changed daily until cell confluency was reached. Media was removed at confluency and cells were left exposed to air, to generate an ALI. The apical surface was washed with 200 μl of sterile PBS to remove mucus and basal media changed every 48 h until the cells underwent mucociliary differentiation. Differentiation of the cells was assessed by, immunofluorescence (IF) using staining for the structural cilia marker β-tubulin, and by RT-PCR for CBE1, SPLUNC1 and MUC5B, markers shown to be upregulated during the process of differentiation.^32^ Cells were used for experiments following culture at ALI for 21 days.

**Generation of HRV stocks.** HRV minor group serotype 1B (HRV-1B) and major group serotype 16 (HRV-16) were obtained from ATCC and viral stocks (average 2.5 × 10^7^ TCID_50_ per ml) generated by infecting HeLa Ohio cells as previously described,^21^ and titered by determining the development of cytopathic effect (CPE).

**Virus CPE assay.** Subconfluent Ohio HeLa cells in 96-well plates were exposed to serial dilutions of infectious supernatants. Development of a CPE was visualized after 4 days. Assays were performed in eight replicate wells, and endpoint titers were defined by the highest dilution at which CPE was observed in 50% of the wells (TCID_50_).

**Preparation of phospholipid liposomes.** The lipids 1-stearoyl-2-arachidonoyl-*sn*-glycero-3-phospho-L-serine, 1-palmitoyl-2-arachidonoyl-*sn*-glycero-3-phosphocholine, 1,2-distearoyl-*sn*-glycero-3-phosphocholine and TopFluor-SAPS were purchased from Avanti Polar Lipids (Alabaster, AL). Liposomes were prepared using the lipid hydration method.^16^ Liposomes were stored at 4 °C in glass vials.

**Liposome characterization.** Liposomal size was measured using photon correlation spectroscopy using a ZetaPlus (Brookhaven Instrument Corporation, Holtsville, NY). The phase transitional behavior of liposomes was studied using a differential scanning calorimeter (Diamond DSC; PerkinElmer, MA). Stability of free and liposomal SAPS was analyzed by direct infusion mass spectrometry (4000 QTRAP LC/MS/MS). Liposomal and free SAPS were diluted in methanol to 100- and 1,000-fold, respectively. Three replicates were prepared for each sample type. These were directly infused at 10 μl per min on a QTRAP mass spectrometer (4000 QTRAP LC/MS/MS), to monitor chemical stability of SAPS by analyzing its molecular mass (810.6 a.m.u., [M−H]^−^) in negative ionization mode. Samples were analyzed weekly over 4 weeks and were stored at 4 °C. The MS conditions were as follows: source temperature 0 °C, ion source gas 1 (GS1) 15, GS2 0, declustering potential (DP) −60 V, entrance potential (EP) −10 V and spray voltage −4.5 kV. Briefly, full scan spectra was acquired in the first quadrupole (Q1) over 500–1,000 a.m.u., at unit mass resolution and profile mode, for 2 s and summed for 10 scans.

**Cell treatment and infection.** Epithelial cells were either pre-treated with liposomes for 4 h before viral infection, or added at 1, 4, or 8 h post infection. Epithelial cells were infected with HRV at the indicated TCID_50_ per ml for 1 h, as described previously,^21, 60^ following which virus was removed, cells were washed, and 1 ml infection medium containing liposomes was then added to the appropriate wells. Epithelial cells stimulated with recombinant cytokine were pre-treated with liposomes for 1 h before the addition of IFN-β (PeproTech EC, London, UK). Liposomes remained present throughout. Cell supernatants, lysates or mRNA were collected at the appropriate time points and stored at −80 °C.

**Transient transfections of BEAS-2B cells with plasmid DNA.** Transient plasmid transfection was carried out as previously described^42^ using constitutively active TRIF, MAVS, or the pUNO1 control vector (Invivogen, France), 0.25 μg CXCL8 or IFN-β reporter, 0.1 μg of Renilla plasmid (Promega, Southampton, UK), and Superfect transfection reagent (Quiagen, Manchester, UK) kindly provided by Dr MR Edwards (Imperial College, London, UK). At 5 h post transfection, cells were washed and treated with SAPS at the indicated concentrations. Lysates were collected at 24 h, and luciferase measured according to the dual luciferase protocol (Promega) using a Berthold luminometer (Berthold Technologies, Herts, UK) with data expressed as ratios of firefly over renilla luciferase.

**RT-PCR and qPCR.** RNA was prepared from cell lysates and converted to cDNA as previously described.^60^ RT-PCR was carried out using GoTaq Flexi DNA polymerase (Promega) and primers specific for TLR3, RIG-I, MDA5, MAVS, IRF1, IRF3, IRF7, CBE1, SPLUNC1, MUC5B, GAPDH, and β-ACTIN. qPCR was carried out as described^12, 21^ using primers and probes specific to HRV, IFN-β, IFN-λ1, and IFN-λ2 (Sigma-Aldrich), and TaqMan gene expression assay probe set specific to GAPDH (Hs00182082_m1) (Applied Biosystems, Paisley, UK). PCR reactions were performed on an ABI7900 TaqMan (Applied Biosystems), with the target genes quantified against a standard curve of plasmids containing known copy numbers of target genes. IFN-β, IFN-λ1, and IFN-λ2 expression was subsequently normalized to GAPDH.

**Immunofluorescence.** For localization studies, BEAS-2B cells were seeded onto collagen-coated microchamber culture slides (MatTek Corporation, Ashland, MA) and grown overnight. Cells were subsequently incubated with TopFluor-SAPS (100 μg ml^−1^) (Avanti Polar Lipids) for the indicated time periods. For studies examining SAPS uptake into differentiated epithelial cells, PBECs were cultured at ALI as described above and subsequently incubated with TopFluor-SAPS (25 μg ml^−1^) (Avanti Polar Lipids) for 30, 60, 120, and 180 min. Cells were then fixed with 4% paraformaldehyde, permeabilized with PBS containing 0.1% saponin and 5% normal goat serum for 60 min, and incubated with primary antibodies (EEA1 and GM130; BD Biosciences, Oxford, UK or β-Tubulin; Sigma-Aldrich) diluted 1:100 in PBS containing 0.1% saponin and 1% bovine serum albumin overnight at 4 °C directly on the microchamber culture slide or transwell membrane. After extensive washing, cells were incubated with AlexaFluor 555-conjugated secondary antibodies diluted 1:1,000 (Molecular Probes, Paisley, UK) for EEA1 and GM130, or AlexaFluor 488-conjugated secondary antibody diluted 1:200 (Molecular Probes) for β-Tubulin at RT for 60 min in the dark. Cellular DNA was stained with 4,6-diamidino-2-phenylindole (DAPI) (Molecular Probes). Cells were washed, mounted with ProLong Antifade Kit (Molecular Probes) and imaged on a Nikon A1 confocal microscope (Nikon Instruments, UK) and viewed using Image J software (Version 1.44o; NIH).

**Western blot.** Western blot analysis was conducted as previously described.^14^ Samples were probed with anti-phospho-STAT1, anti-STAT1 total (Abcam, Cambridge, UK), or anti-actin (Sigma-Aldrich). Densitometric analysis was carried out using Image J software (Version 1.44o; NIH).

**Statistical analysis.** All data are presented as mean±s.e.m. (where appropriate) of at least three independent experiments. Data were analyzed using the statistical tests stated using Prism 6.0 software (GraphPad Inc., La Jolla, CA). Multiple comparisons were performed by using two-way ANOVA and Bonferroni’s post test. Experiments using transfection with plasmids were analyzed by one-way ANOVA and Tukey’s post test. Significant differences are indicated by by *P<0.05, **P<0.01 ***P<0.001, and ****P<0.0001.

## Supplementary information

Supplementary Information (DOC 44 kb)

Supplementary Information (DOC 4439 kb)

## PowerPoint slides

PowerPoint slide for Fig. 1
PowerPoint slide for Fig. 2
PowerPoint slide for Fig. 3
PowerPoint slide for Fig. 4
PowerPoint slide for Fig. 5
PowerPoint slide for Fig. 6
PowerPoint slide for Fig. 7
PowerPoint slide for Fig. 8
PowerPoint slide for Fig. 9
PowerPoint slide for Fig. 10
