# Supplementary material for: Human rhinovirus-induced inflammatory responses are inhibited by phosphatidylserine containing liposomes
Source: Mucosal Immunol. 2016 Feb 24;9(5):1303–16. doi: 10.1038/mi.2015.137 (PMC4883656; doi:10.1038/mi.2015.137)
Supplement: Supplementary file 1 — Supplementary Information (DOC 44 kb) [file 41385_2016_BFmi2015137_MOESM433_ESM.doc]

Human Rhinovirus Induced Inflammatory Responses are inhibited by Phosphatidylserine Containing Liposomes

CA Stokes, R Kaur, MR Edwards, M Mondhe, D Robinson, EC Prestwich, RD Hume, C Marshall, Y Perrie, VB O’Donnell, JL Harwood, I Sabroe and LC Parker

**Online supplementary materials**

**Supplementary Table S1:** Vesicle size (nm) of SAPS, SAPS+25% DSPC and PAPC formulations stored at 4°C was measured in PBS buffer using a Brookhaven ZetaPlus instrument at the time intervals indicated.

| **Formulation** | **Size (nm)** | **Polydispersity Index (PI)** | **Transition Temperature (°C)** |
| --- | --- | --- | --- |
| SAPS | 203.6±48.0 | 0.279±0.04 | 17.48 |
| SAPS+25%DSPC | 264.3±36.2 | 0.335±0.022 | 18.19 |
| PAPC | 629.0±90.7 | 0.519±0.15 | 16.67 |
