# Supplementary material for: Human rhinovirus-induced inflammatory responses are inhibited by phosphatidylserine containing liposomes
Source: Mucosal Immunol. 2016 Feb 24;9(5):1303–16. doi: 10.1038/mi.2015.137 (PMC4883656; doi:10.1038/mi.2015.137)
Supplement: Supplementary file 2 — Supplementary Information (DOC 4439 kb) [file 41385_2016_BFmi2015137_MOESM434_ESM.doc]

Human Rhinovirus Induced Inflammatory Responses are inhibited by Phosphatidylserine Containing Liposomes

CA Stokes, R Kaur, MR Edwards, M Mondhe, D Robinson, EC Prestwich, RD Hume, C Marshall, Y Perrie, VB O’Donnell, JL Harwood, I Sabroe and LC Parker

**Online supplementary materials**

**Supplementary Figure S1:** Vesicle size (nm) of 1-stearoyl-2-arachidonoyl-*sn*-glycero-3-phospho-L-serine (SAPS), SAPS+25% 1,2-distearoyl-*sn*-glycero-3-phosphocholine (DSPC) and 1-palmitoyl-2-arachidonoyl-*sn*-glycero-3-phosphocholine (PAPC) formulations stored at 4°C was measured in PBS buffer using a Brookhaven ZetaPlus instrument at the time intervals indicated.

**Supplementary Figure S2:** The phase transitional behaviour of liposomes was studied using a differential scanning calorimeter (DSC). SAPS (i), SAPS+25% DSPC (ii) and PAPC (iii) formulations.

**Supplementary Figure S3:** Analysis of SAPS using LC/MS/MS*.* A-C: Free SAPS and SAPS liposomes were separated at weekly intervals over the time period indicated using a QTRAP mass spectrometer to monitor chemical stability of SAPS by analysing its molecular mass (810.6 amu, [M-H]^-^) in negative ionisation mode. (**a-b**): Scans collected at week 0 and week 4.

**Supplementary Figure S4:** Time course of SAPS modulation of human rhinovirus (HRV)-induced cytokine production. BEAS-2B cells infected with HRV (MOI 3/TCID_50_/ml 1x10^7^) for 1hr, then treated with SAPS at the doses indicated. At the time-points indicated, CXCL8 (**a, b**), CCL5 (**c, d**) or CXCL10 (**e, f**) release were measured, and IFN-β mRNA expression quantified and presented as total IFN-β mRNA copies per 1μg RNA normalized to GAPDH expression (**g, h**). Data shown are mean ± SEM (*n = 2).*

**Supplementary Figure S5:** Addition of SAPS 4h prior to HRV infection moderately effects HRV-16-induced cytokine production. BEAS-2B cells were pretreated with SAPS for 4h prior to HRV infection. Cells were infected with HRV (MOI 3/TCID_50_/ml 1x10^7^) for 1h, then washed and incubated for 24h (**a, c**) or 48h (**b, d**). Cell-free supernatants were prepared and CXCL8 (**a, b**) and CCL5 (**c, d**) release were measured. Data shown are mean ± SEM (*n = 3).* Significant differences are indicated by *.

**Supplementary Figure S6:** Modulation of the IFNAR activity by PAPC was assessed. BEAS-2B cells were with HRV (MOI 3/TCID_50_/ml 1x10^7^), (a-c), followed by treatment with SAPS; or pre-treated with SAPS for 1hr prior to the addition of IFN-β (10 and 100ng/ml)(d-f). Whole-cell lysates were collected at 24h. A representative blot of 3 (c) or 4 (f) independent experiments is shown. Data shown are mean ± SEM (*n=1* for panel a, b) and *n=4* for panel d, e). Significant differences are indicated by **, *P* < 0.01, ***, *P* < 0.001 and ****, *P* < 0.0001.

**Supplementary Figure S7:** SAPS modestly effects viral replication over time. BEAS-2B cells were infected with HRV (MOI 3/TCID_50_/ml 1x10^7^) for 1hr, then treated with SAPS at the doses indicated. At the time-points indicated, intracellular viral RNA expression was quantified with data presented as the total intracellular viral RNA copies per 1μg RNA. Data shown are mean ± SEM (*n =* 2).

**Supplementary Figure S8:** SAPS does not alter pattern recognition receptor (PRR) expression following HRV infection. BEAS-2B cells were infected with HRV (MOI 3/TCID_50_/ml 1x10^7^), followed by SAPS treatment at concentrations of 10μg/ml (i) or 50μg/ml (ii). PRR expression was explored at 24h. Glyceraldehyde-3-phosphate dehydrogenase (GAPDH) was used as a loading control. A representative image of *n=3* independent experiments is shown.

**Supplementary Figure S9: SAPS does not significantly alter interferon regulatory factor (IRF) expression following HRV infection.** BEAS-2B cells were infected with HRV (MOI 3/TCID_50_/ml 1x10^7^), followed by SAPS treatment at concentrations of 10μg/ml (i) or 50μg/ml (ii). After 24h, IRF expression was explored. GAPDH was used as loading control. A representative image of *n=3* independent experiments is shown.

**Supplementary Figure S10:** Confirmation of differentiation of ALI cultures. PBECs were differentiated at ALI over 21 days. (a) Expression of the differentiation markers CBE1, SPLUNC1 and MUC5B were analyzed by RT-PCR at day 0, 3 and 21. β-ACTIN was used as loading control. **(b)** A 21 day ALI culture was stained for β-tubulin as outlined in the methods section. The positive green staining shows the presence of cilia following differentiation. DAPI staining is shown in blue. Images were collected using a Nikon confocal microscope and a representative image of 1 independent experiment is shown of stack 3/20 illustrating that positive cilia staining is located at the apical cell surface.

**Supplementary Figure S11:** SAPS can be taken up by differentiated bronchial epithelial cells. A day 21 ALI culture was incubated with TopFluor-SAPS (25μg/ml) for various time periods. Uptake of SAPS to intracellular regions at 180 minutes is shown in green. Early endosomes are shown in red (EEA1). DAPI staining is shown in blue. Z stack images were collected using a Nikon confocal microscope and a representative image is shown of stack 10/20 of 1 independent experiment.


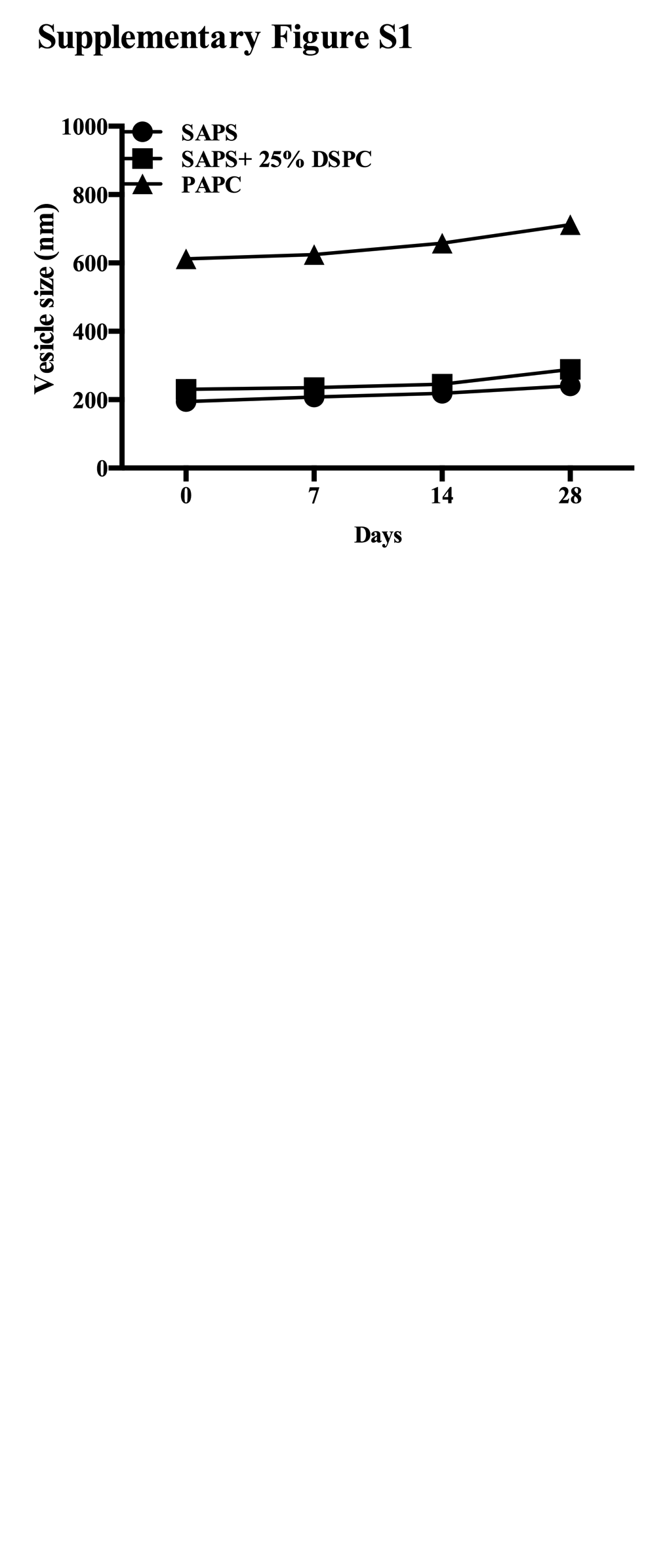


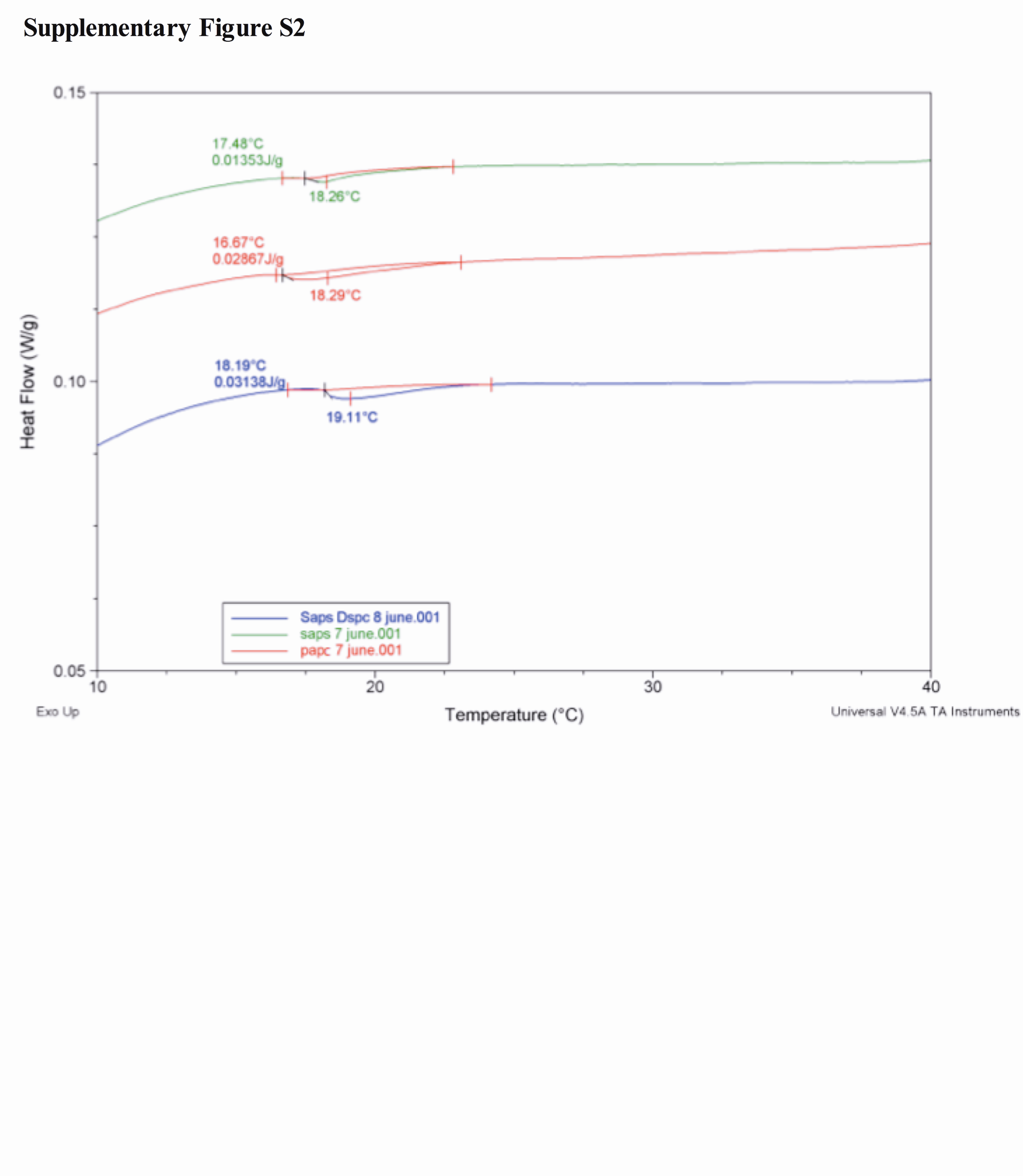


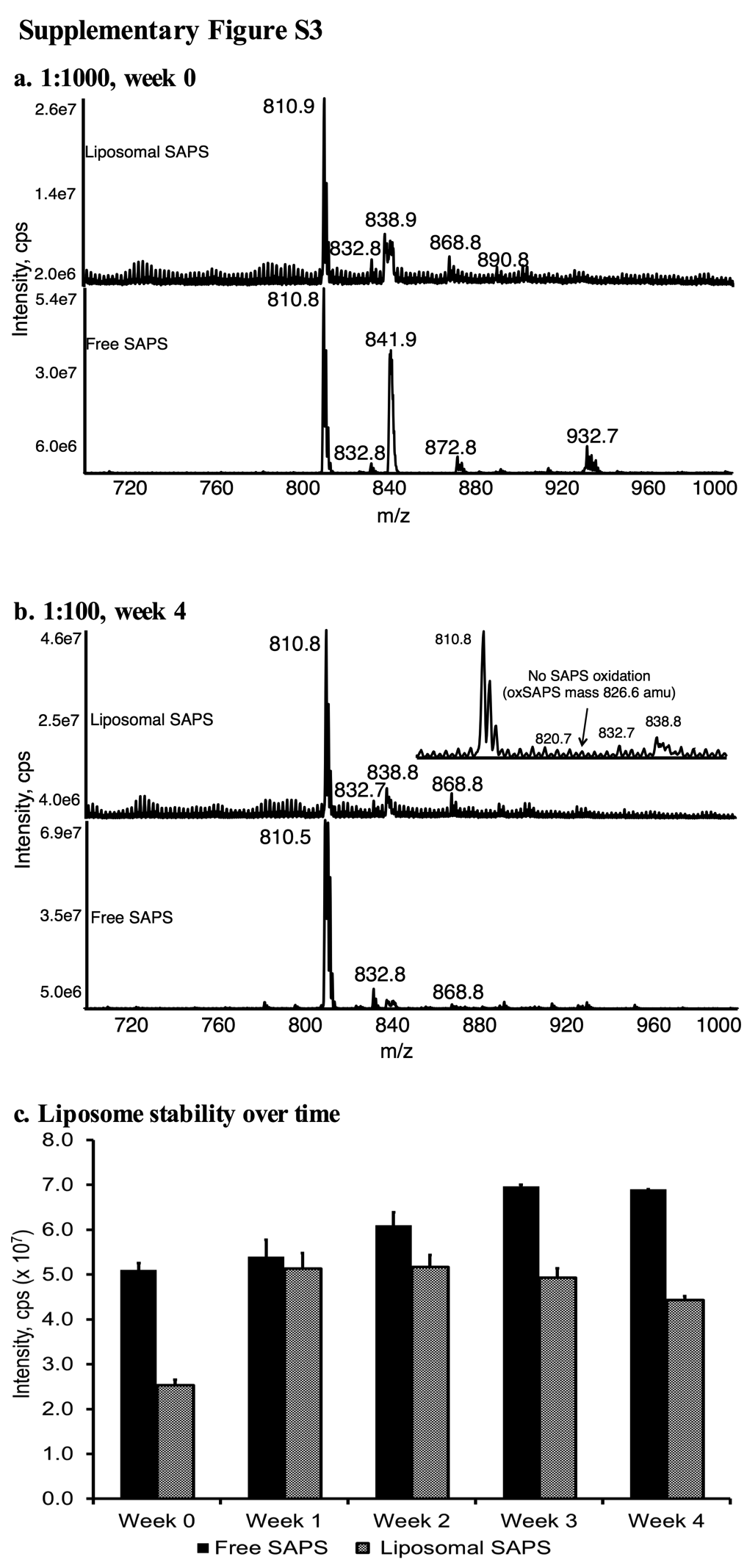


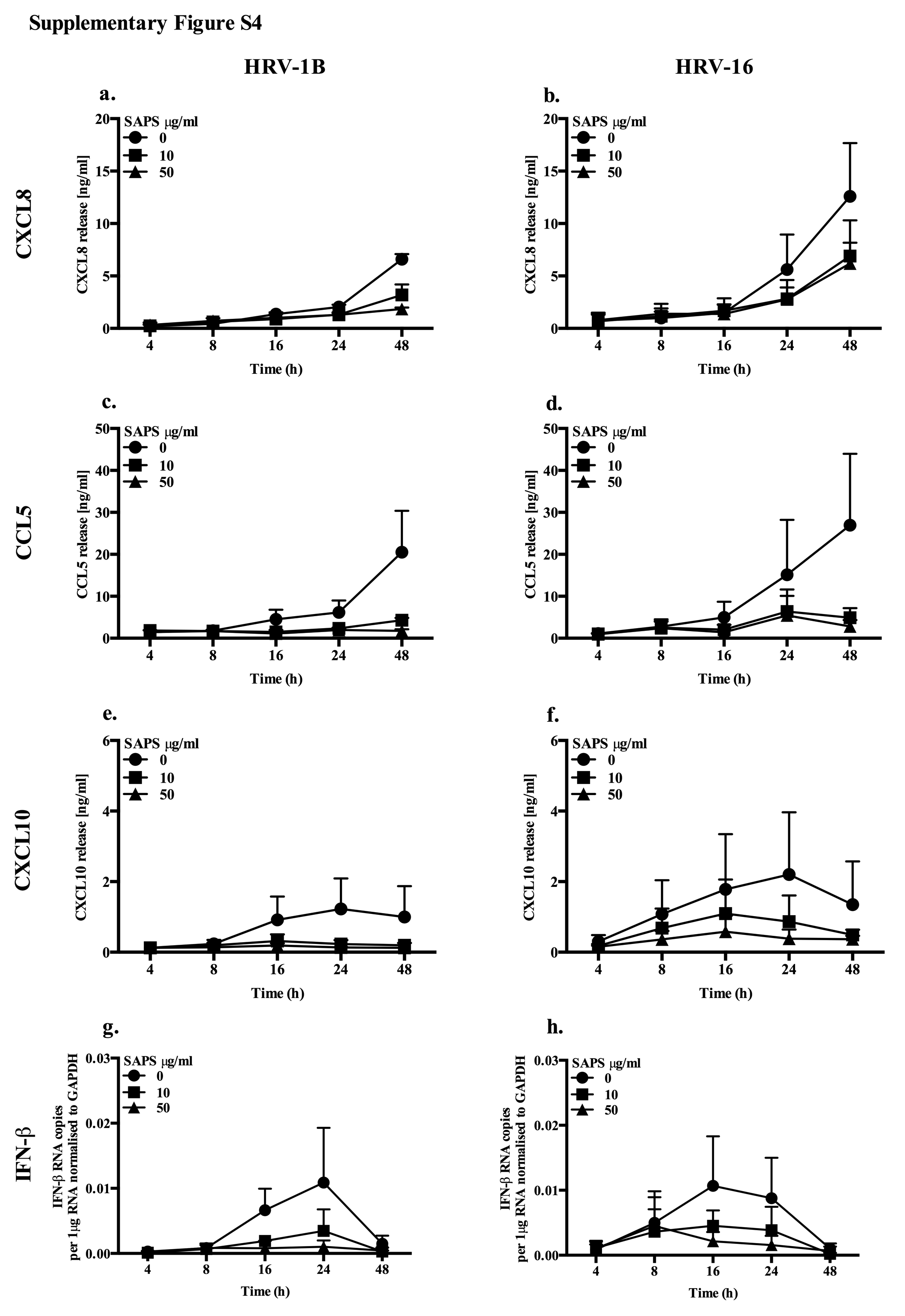


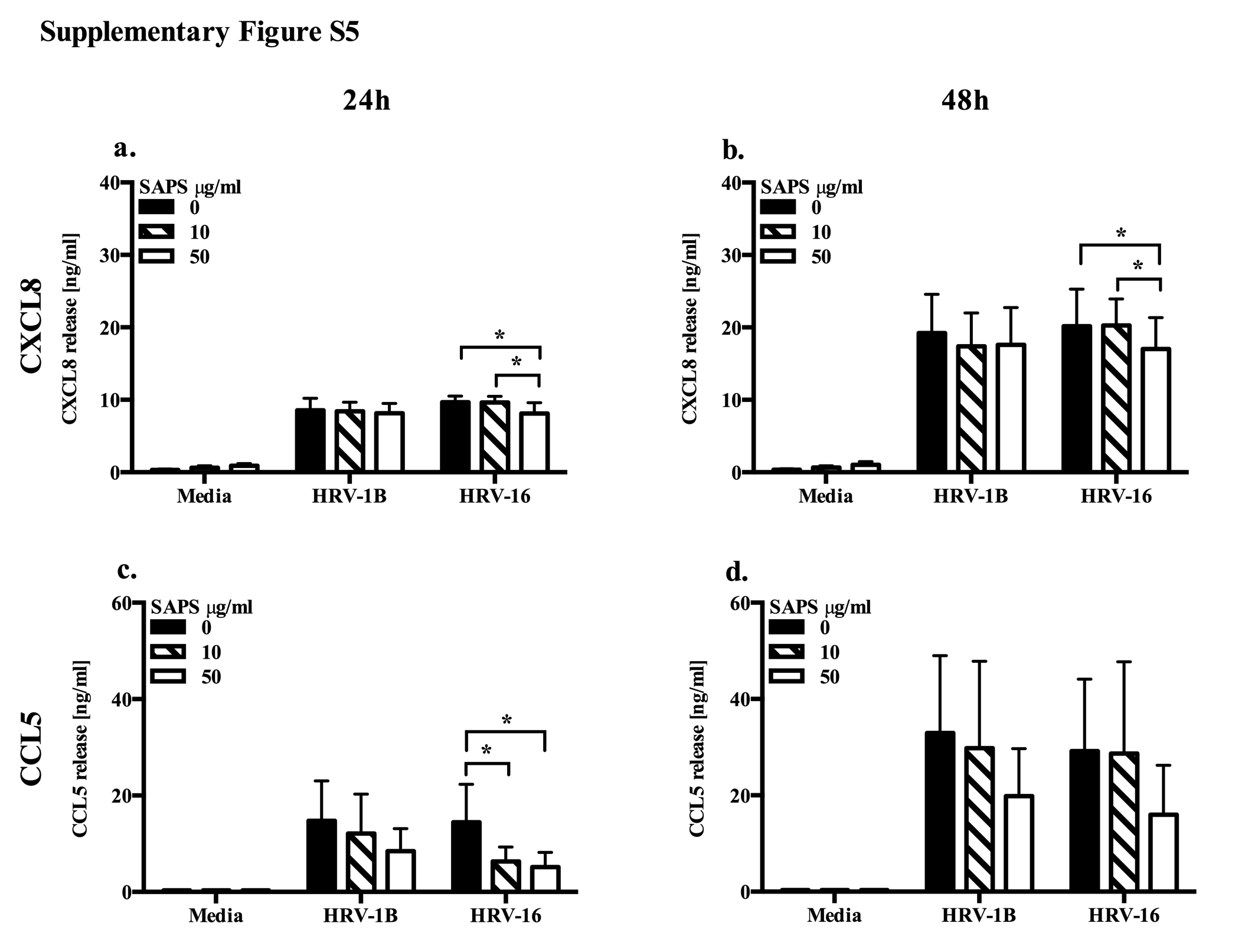


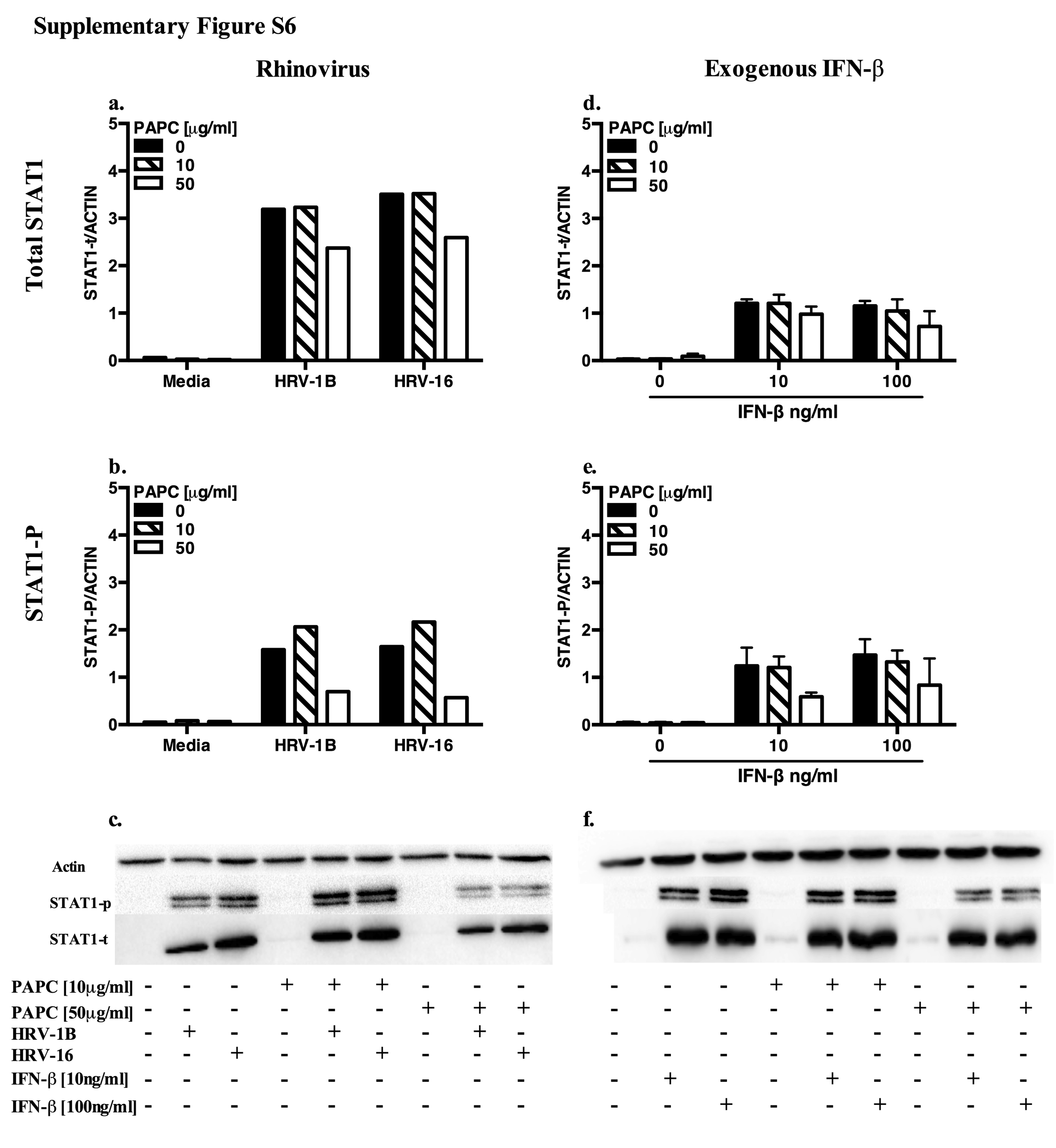


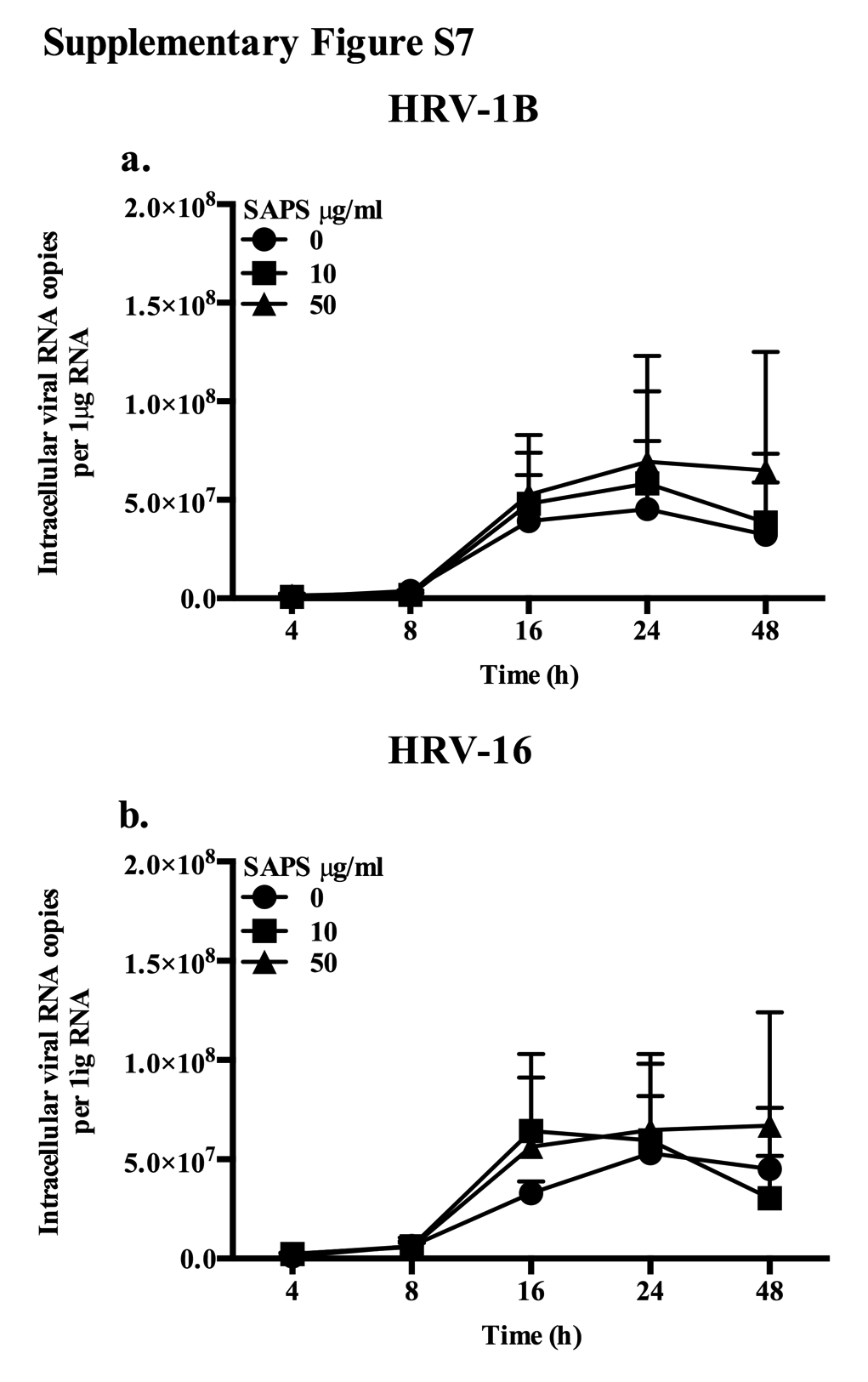


**
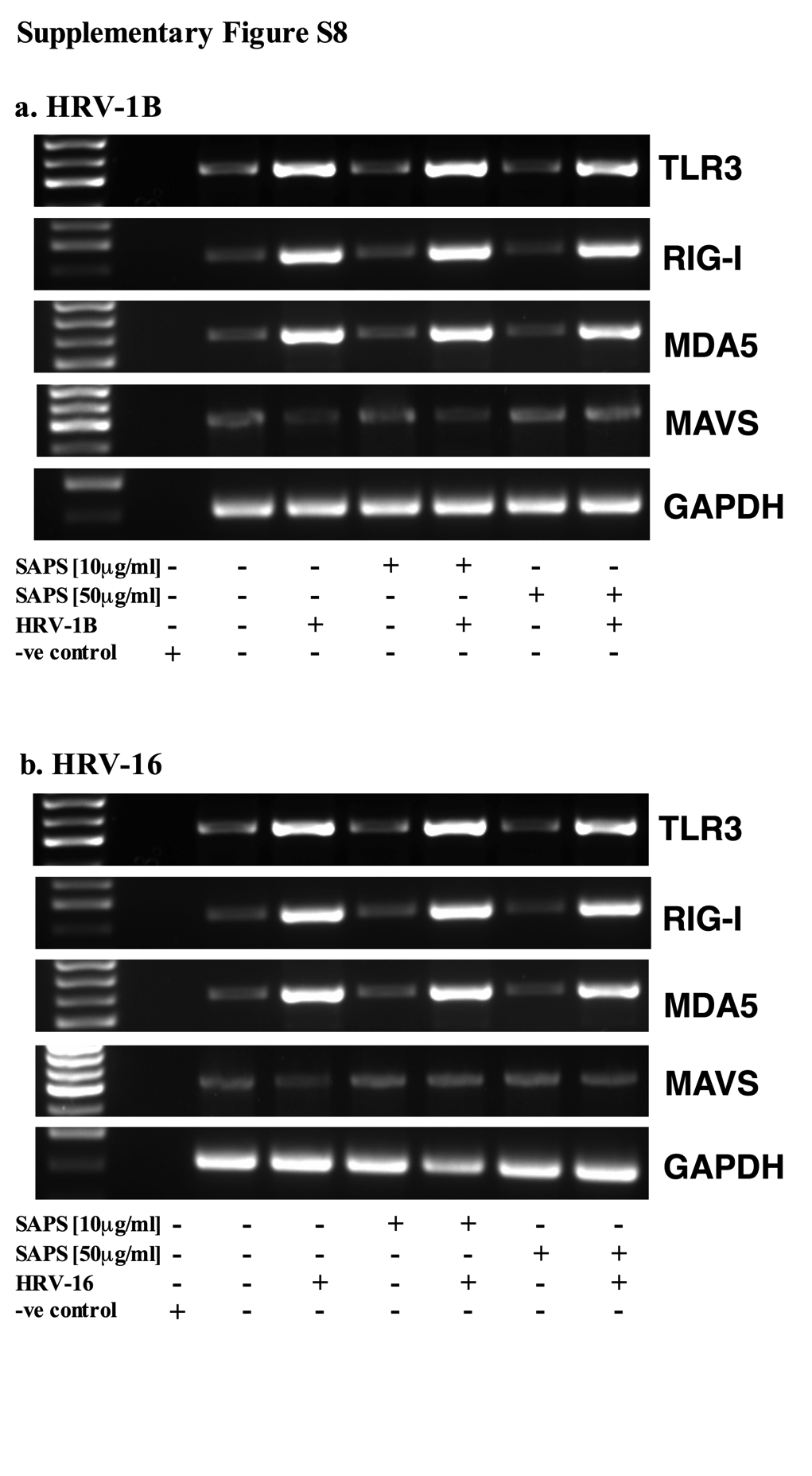
**

**
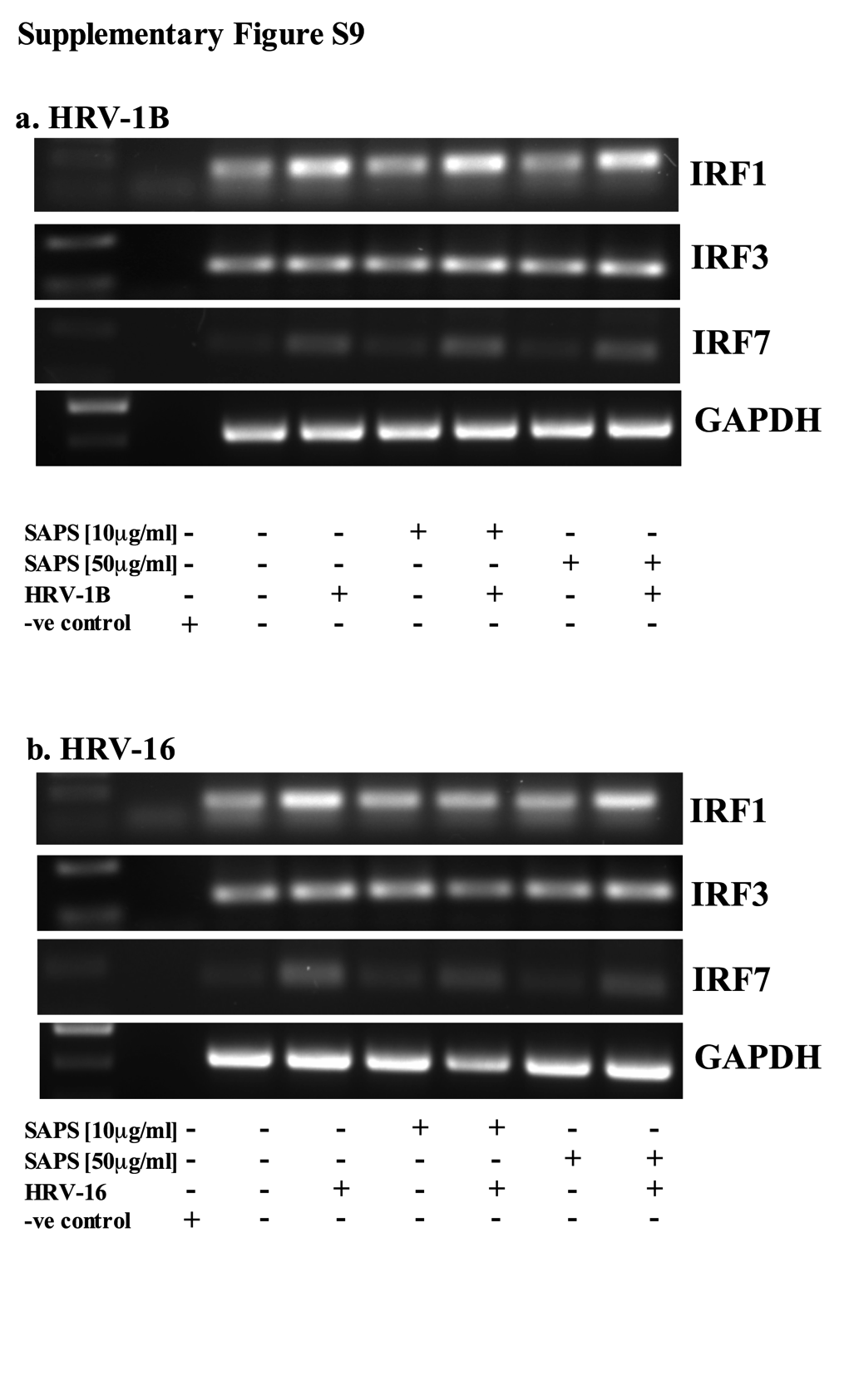
**

**
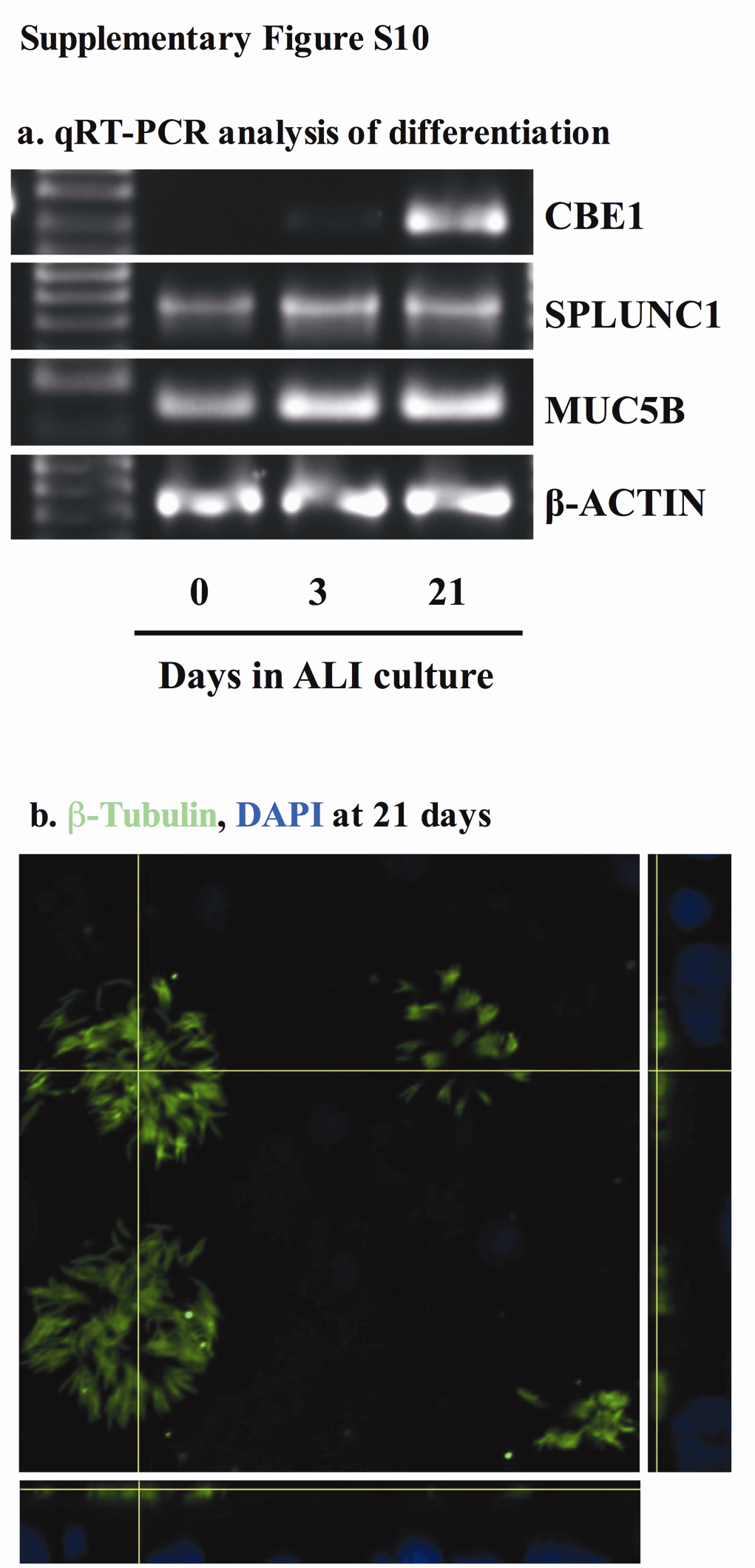
**

**
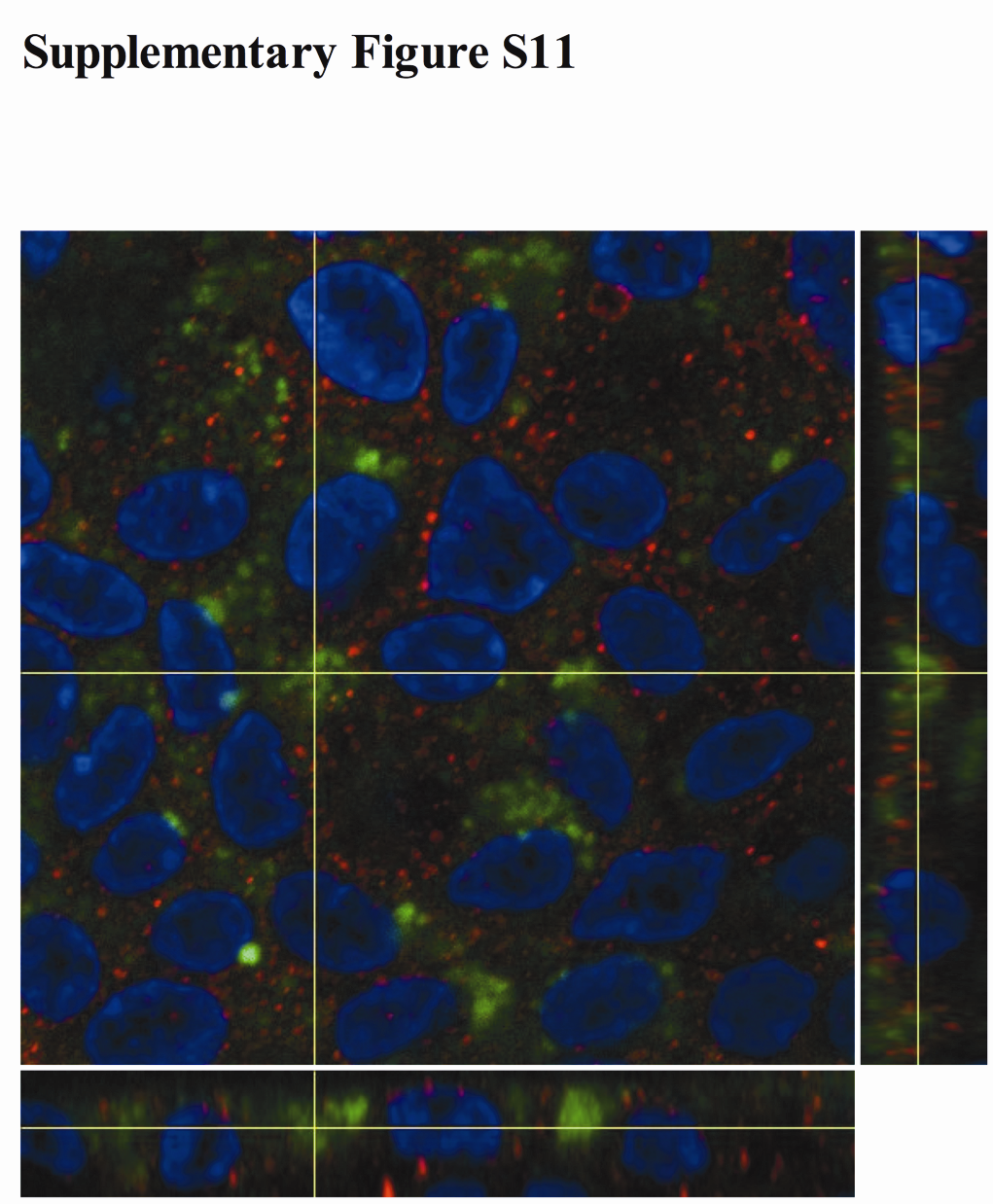
**
