# Supplementary figures and images for: Human rhinovirus-induced inflammatory responses are inhibited by phosphatidylserine containing liposomes
Source: Mucosal Immunol. 2016 Feb 24;9(5):1303–16. doi: 10.1038/mi.2015.137 (PMC4883656; doi:10.1038/mi.2015.137)

## Slide 1
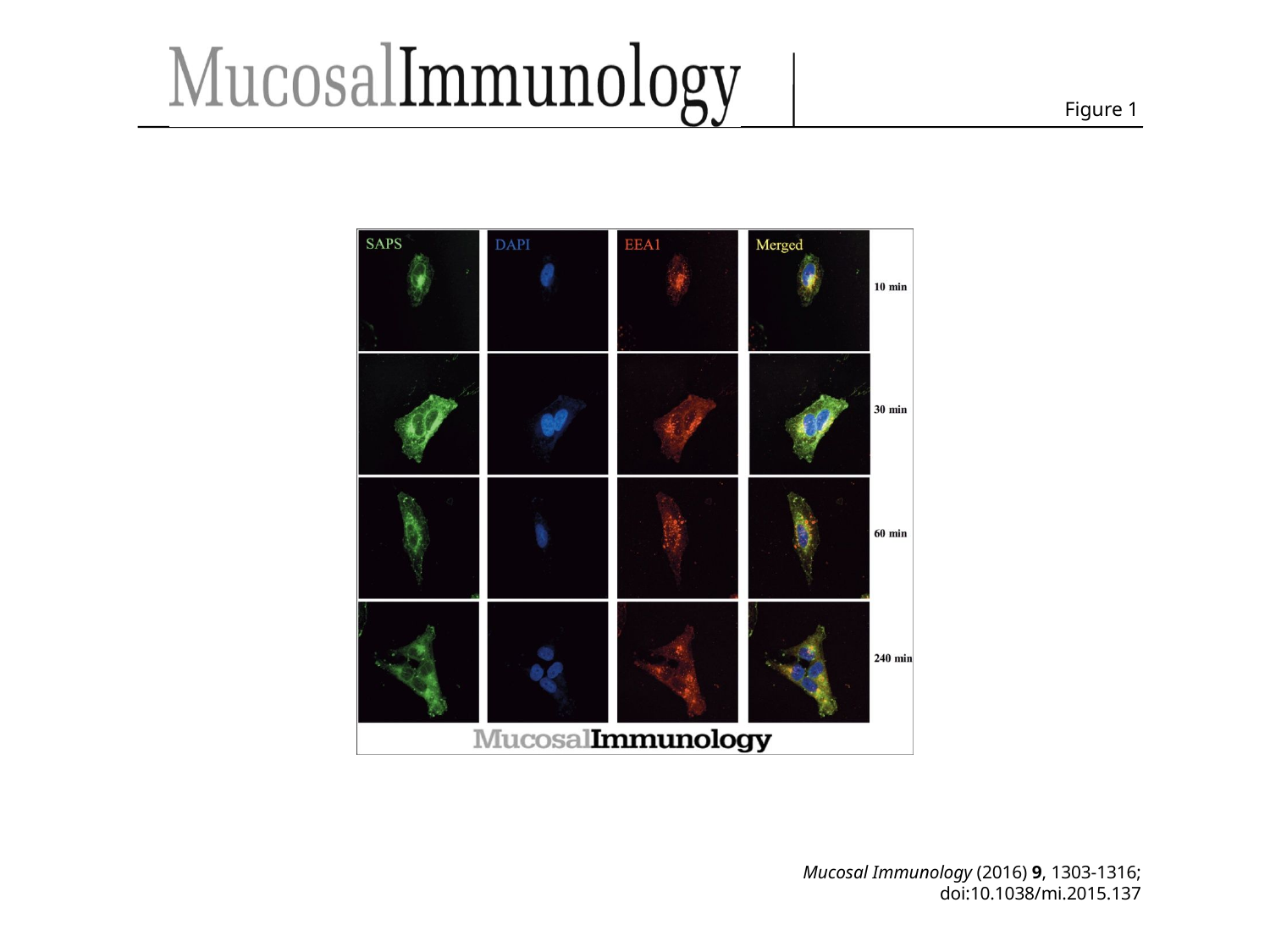

Figure 1
Mucosal Immunology (2016) 9, 1303-1316;
doi:10.1038/mi.2015.137

Supplement: Supplementary file 3 — PowerPoint slide for Fig. 1 [file 41385_2016_BFmi2015137_MOESM423_ESM.ppt]

## Slide 1
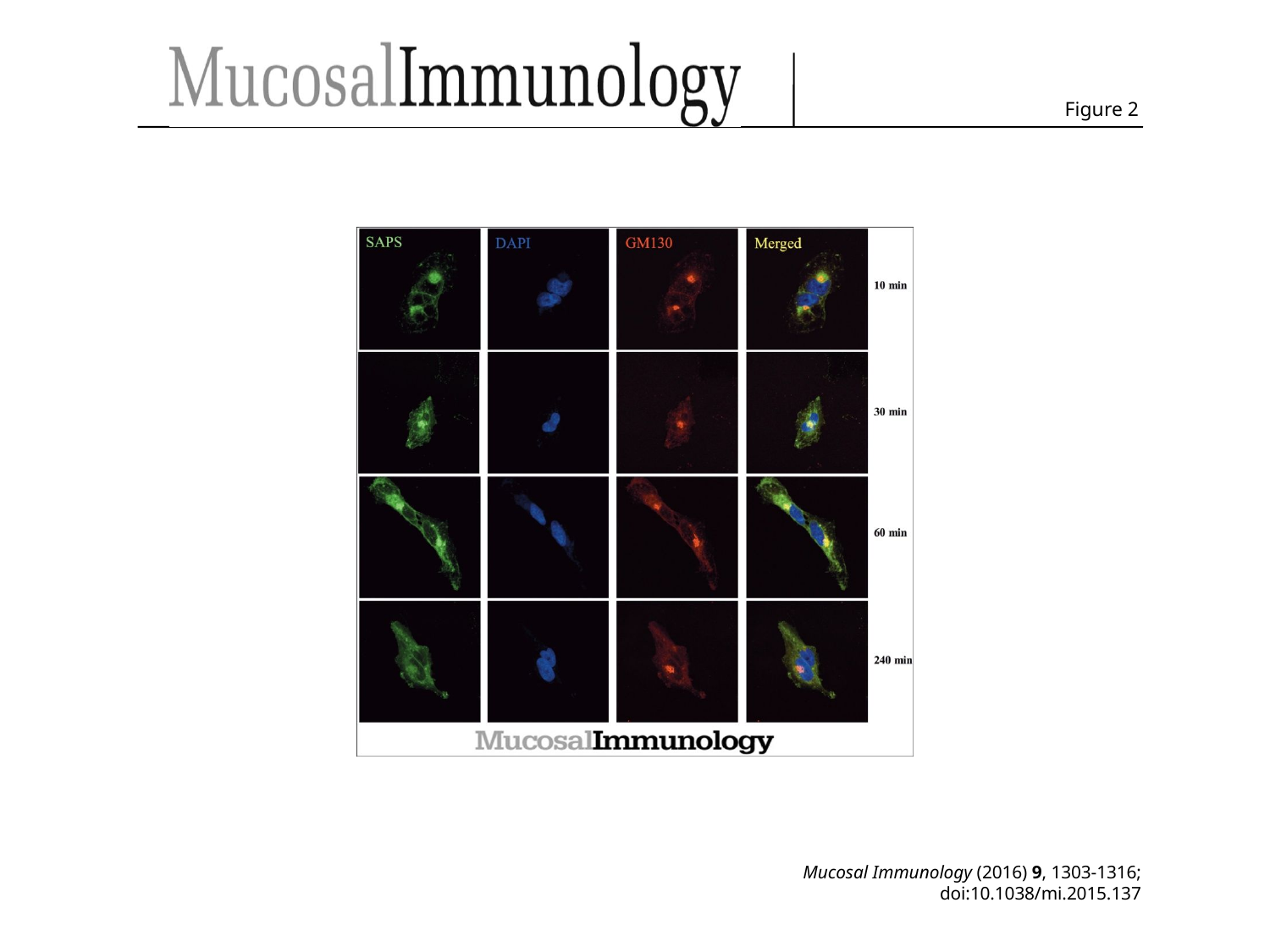

Figure 2
Mucosal Immunology (2016) 9, 1303-1316;
doi:10.1038/mi.2015.137

Supplement: Supplementary file 4 — PowerPoint slide for Fig. 2 [file 41385_2016_BFmi2015137_MOESM424_ESM.ppt]

## Slide 1
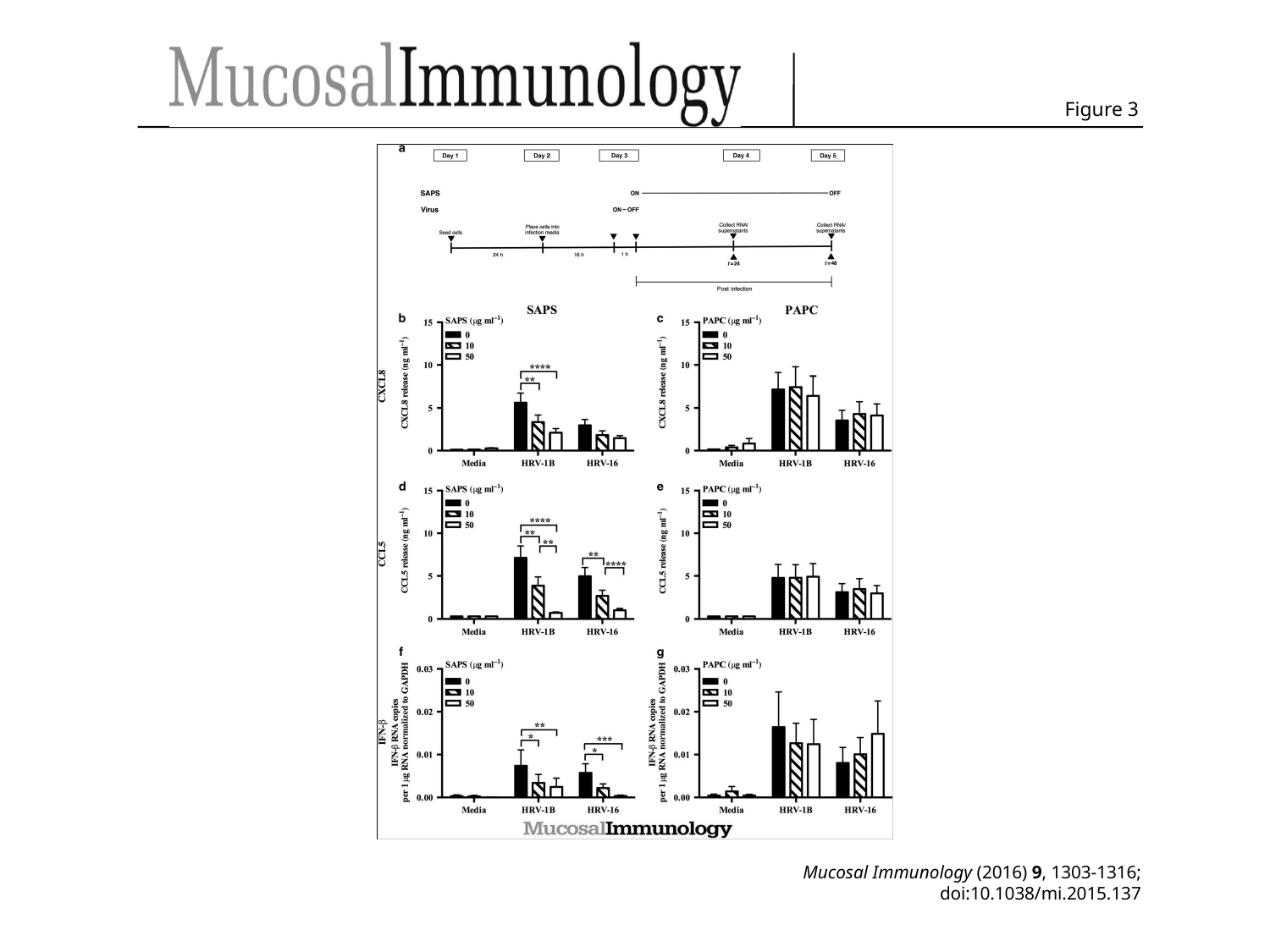

Figure 3
Mucosal Immunology (2016) 9, 1303-1316;
doi:10.1038/mi.2015.137

Supplement: Supplementary file 5 — PowerPoint slide for Fig. 3 [file 41385_2016_BFmi2015137_MOESM425_ESM.ppt]

## Slide 1
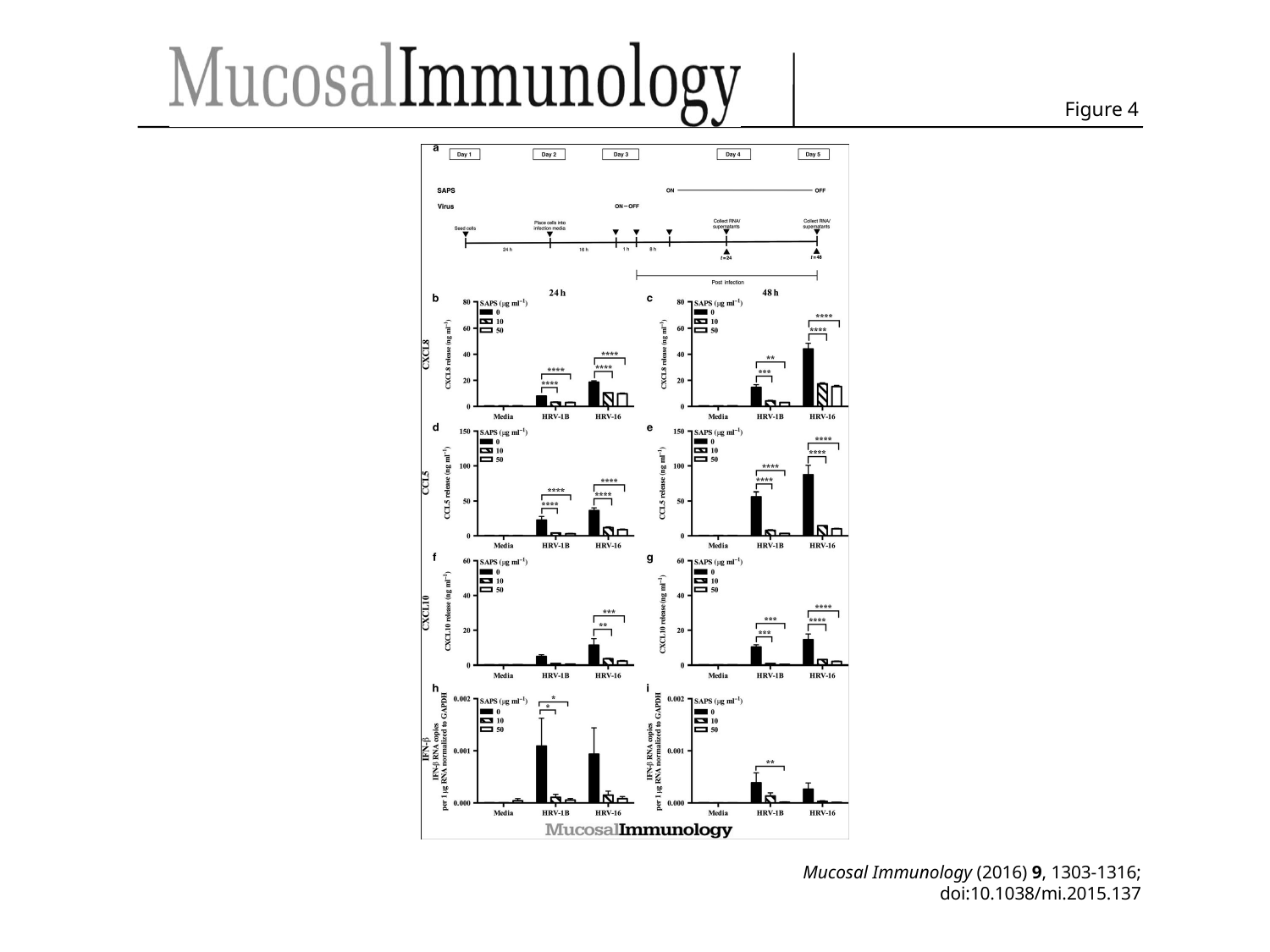

Figure 4
Mucosal Immunology (2016) 9, 1303-1316;
doi:10.1038/mi.2015.137

Supplement: Supplementary file 6 — PowerPoint slide for Fig. 4 [file 41385_2016_BFmi2015137_MOESM426_ESM.ppt]

## Slide 1
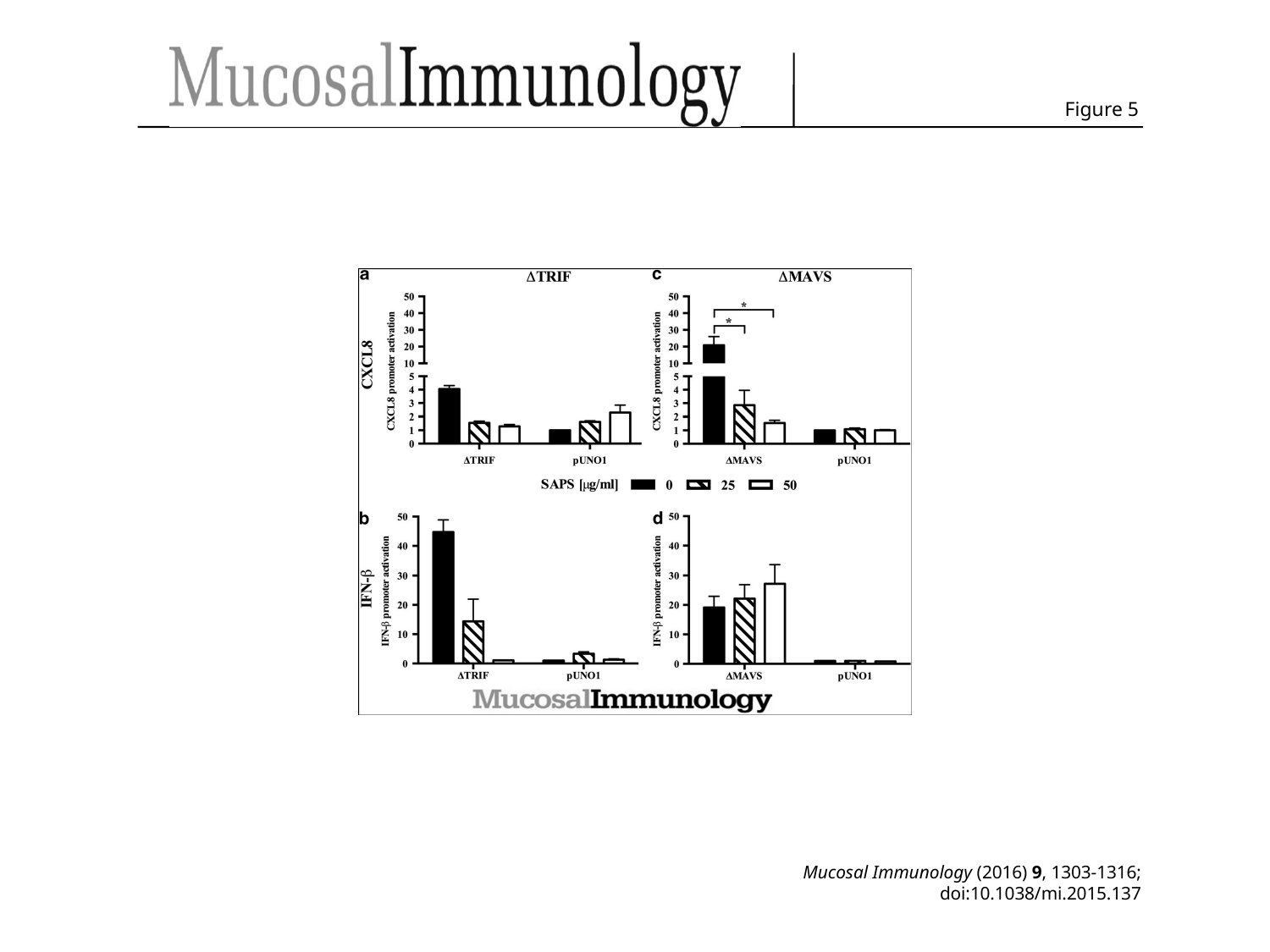

Figure 5
Mucosal Immunology (2016) 9, 1303-1316;
doi:10.1038/mi.2015.137

Supplement: Supplementary file 7 — PowerPoint slide for Fig. 5 [file 41385_2016_BFmi2015137_MOESM427_ESM.ppt]

## Slide 1
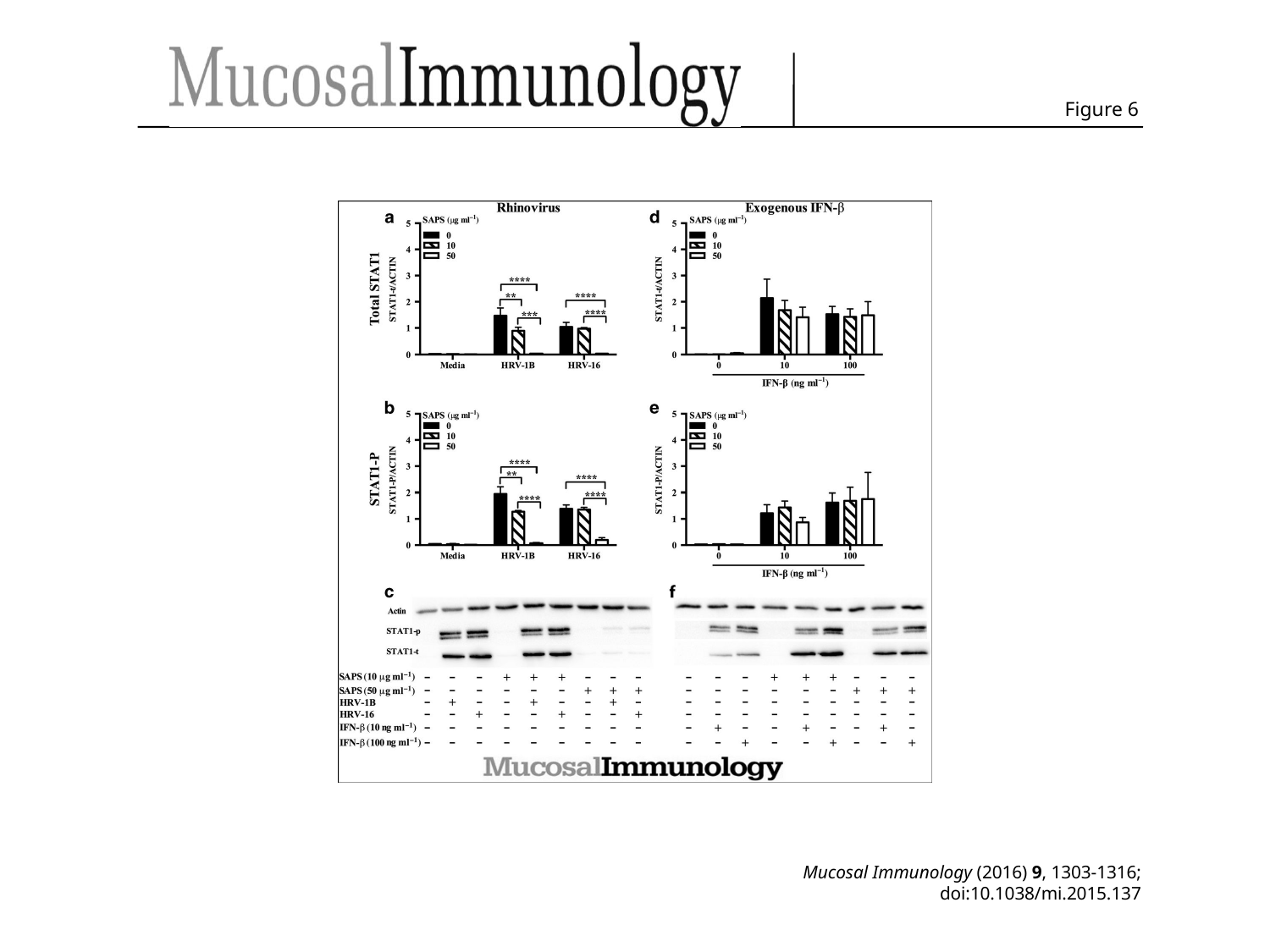

Figure 6
Mucosal Immunology (2016) 9, 1303-1316;
doi:10.1038/mi.2015.137

Supplement: Supplementary file 8 — PowerPoint slide for Fig. 6 [file 41385_2016_BFmi2015137_MOESM428_ESM.ppt]

## Slide 1
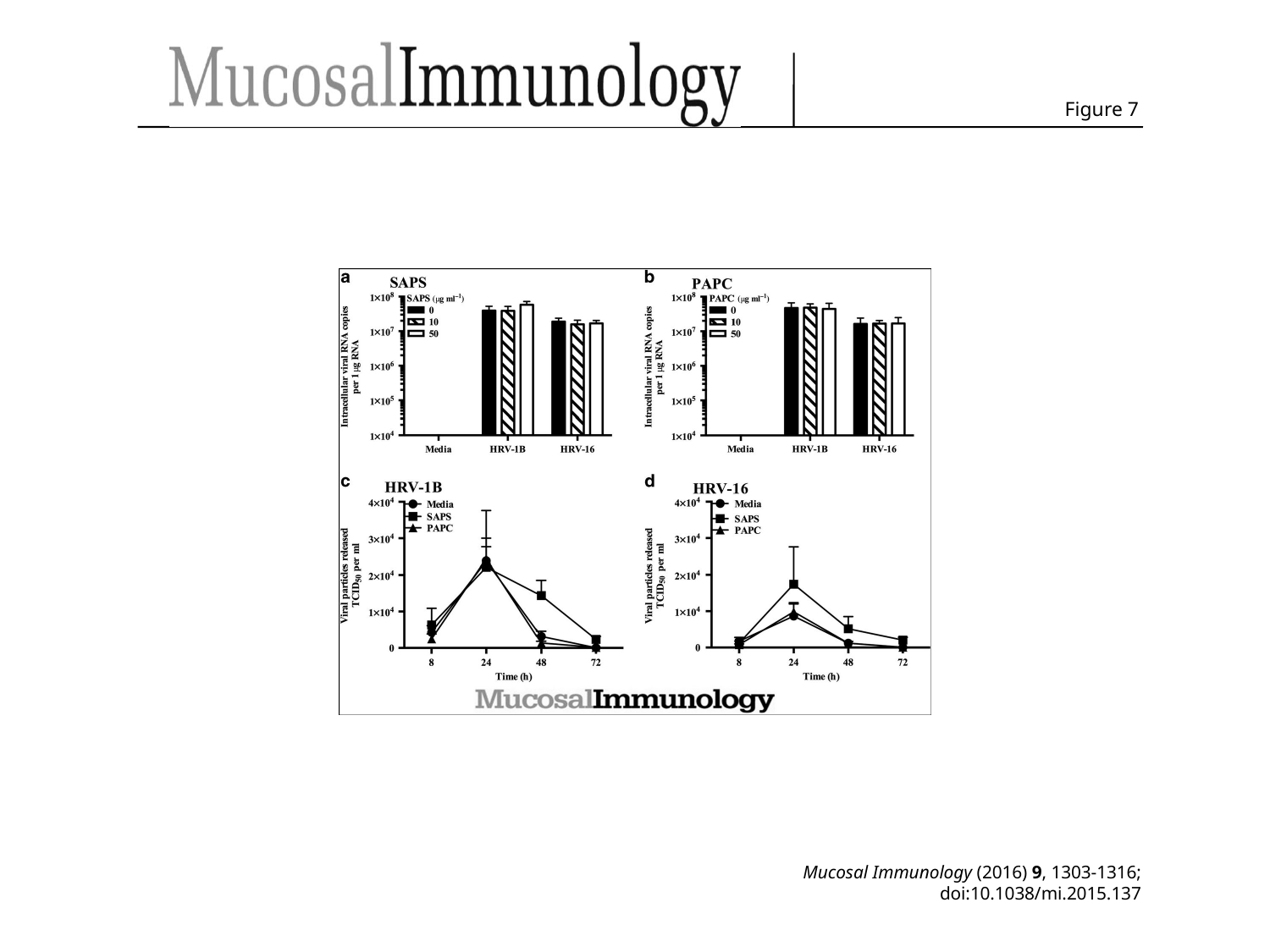

Figure 7
Mucosal Immunology (2016) 9, 1303-1316;
doi:10.1038/mi.2015.137

Supplement: Supplementary file 9 — PowerPoint slide for Fig. 7 [file 41385_2016_BFmi2015137_MOESM429_ESM.ppt]

## Slide 1
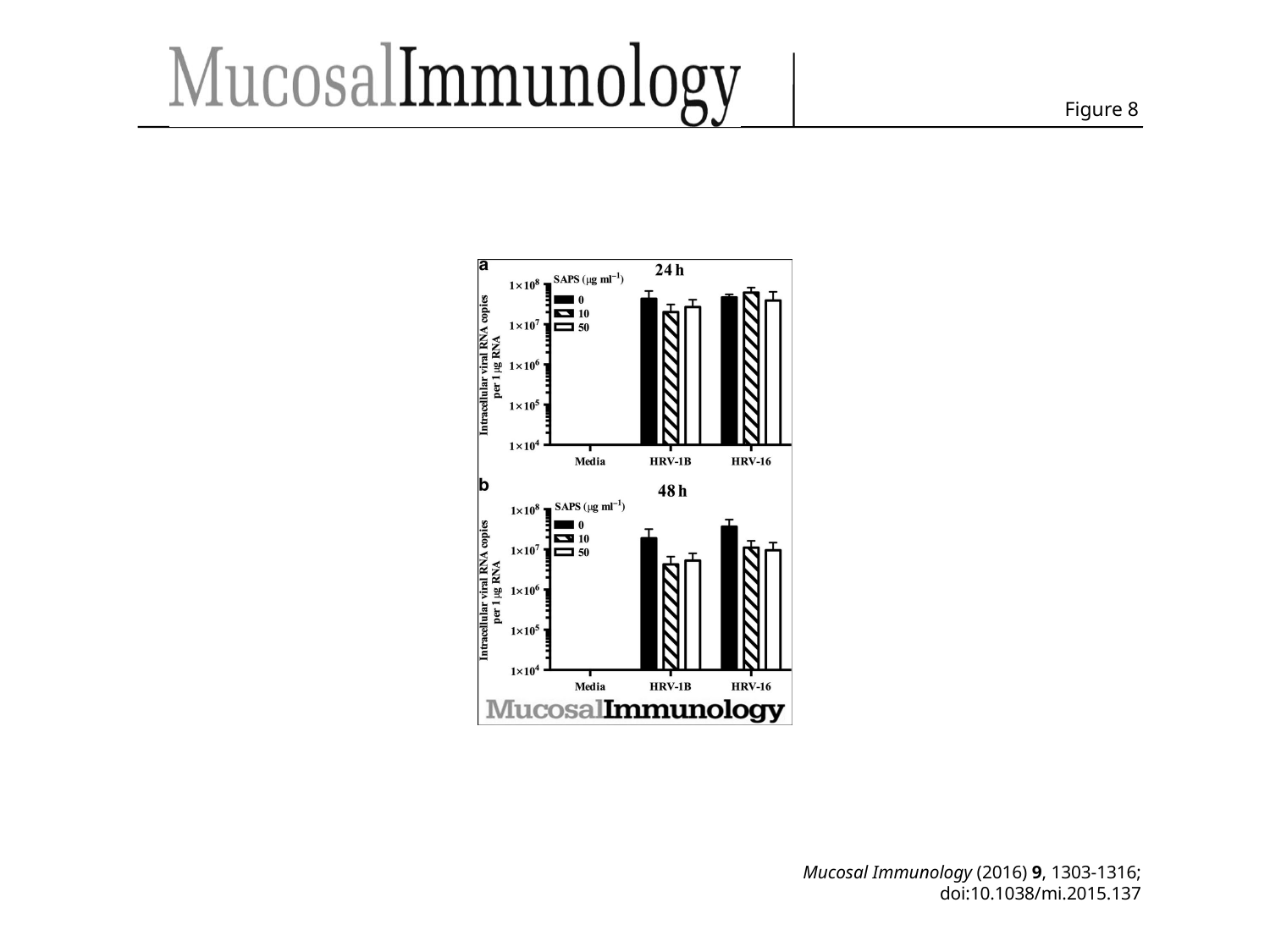

Figure 8
Mucosal Immunology (2016) 9, 1303-1316;
doi:10.1038/mi.2015.137

Supplement: Supplementary file 10 — PowerPoint slide for Fig. 8 [file 41385_2016_BFmi2015137_MOESM430_ESM.ppt]

## Slide 1
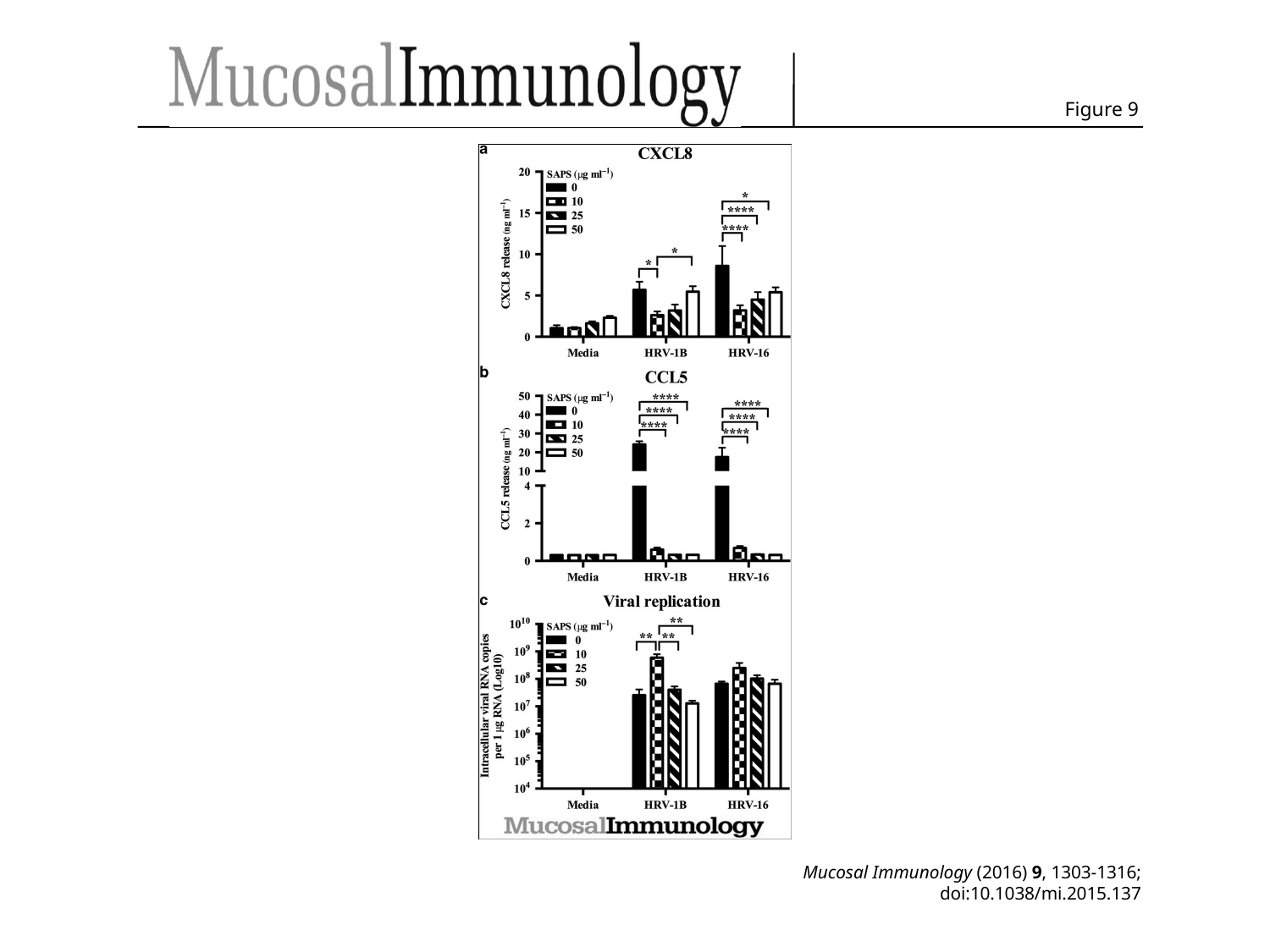

Figure 9
Mucosal Immunology (2016) 9, 1303-1316;
doi:10.1038/mi.2015.137

Supplement: Supplementary file 11 — PowerPoint slide for Fig. 9 [file 41385_2016_BFmi2015137_MOESM431_ESM.ppt]

## Slide 1
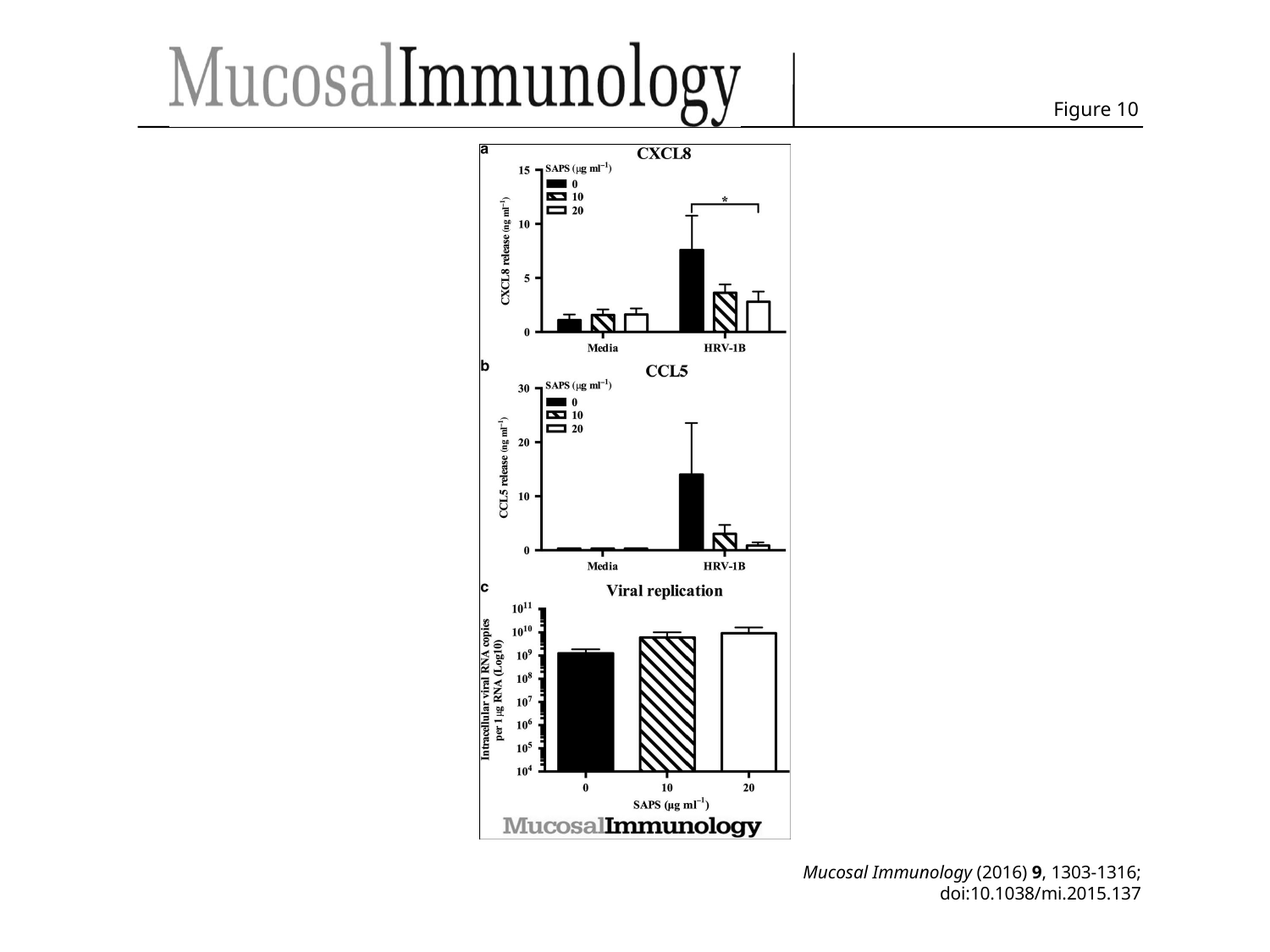

Figure 10
Mucosal Immunology (2016) 9, 1303-1316;
doi:10.1038/mi.2015.137

Supplement: Supplementary file 12 — PowerPoint slide for Fig. 10 [file 41385_2016_BFmi2015137_MOESM432_ESM.ppt]
